# Acetyl-CoA-mediated activation of *Mycobacterium tuberculosis* isocitrate lyase 2

**DOI:** 10.1038/s41467-019-12614-7

**Published:** 2019-10-11

**Authors:** Ram Prasad Bhusal, Wanting Jiao, Brooke X. C. Kwai, Jóhannes Reynisson, Annabelle J. Collins, Jonathan Sperry, Ghader Bashiri, Ivanhoe K. H. Leung

**Affiliations:** 1 School of Chemical Sciences, The University of Auckland, Private Bag 92019, Victoria Street West, Auckland, 1142 New Zealand; 2 Ferrier Research Institute, Victoria University of Wellington, PO Box 600, Wellington, 6140 New Zealand; 3 Maurice Wilkins Centre for Molecular Biodiscovery, The University of Auckland, Private Bag 92019, Victoria Street West, Auckland, 1142 New Zealand; 4 School of Pharmacy and Bioengineering, Hornbeam Building, Keele University, Keele, Staffordshire ST5 5BG UK; 5 School of Biological Sciences, The University of Auckland, Private Bag 92019, Victoria Street West, Auckland, 1142 New Zealand

**Keywords:** Biochemistry, Enzyme mechanisms, SAXS, X-ray crystallography

## Abstract

Isocitrate lyase is important for lipid utilisation by *Mycobacterium tuberculosis* but its ICL2 isoform is poorly understood. Here we report that binding of the lipid metabolites acetyl-CoA or propionyl-CoA to ICL2 induces a striking structural rearrangement, substantially increasing isocitrate lyase and methylisocitrate lyase activities. Thus, ICL2 plays a pivotal role regulating carbon flux between the tricarboxylic acid (TCA) cycle, glyoxylate shunt and methylcitrate cycle at high lipid concentrations, a mechanism essential for bacterial growth and virulence.

## Abstract (web summary)

Isocitrate lyase (ICL) isoforms 1 and 2 are enzymes in the glyoxylate and methylcitrate cycles that enable *Mycobacterium tuberculosis* (*Mtb*) to use lipids as a carbon source. Here the authors present the ligand-free *Mtb* ICL2 and acetyl-CoA bound ICL2 crystal structures, which reveal a structural reorganisation upon acetyl-CoA binding that leads to an activation of its isocitrate lyase and methylcitrate lyase activities.

## Introduction

The ability of *Mycobacterium tuberculosis* (*Mtb*) to preferentially utilise lipids as its carbon source is a metabolic feature that enables chronic infection^1–4^. Isocitrate lyase (ICL) isoforms 1 and 2 (Supplementary Fig. 1) are key enzymes in this process, through their roles in the glyoxylate and methylcitrate cycles (Supplementary Fig. 2)^5,6^. Both enzymes are essential for in vivo growth and virulence^7,8^, but most studies have focused on the roles and structure of ICL1^6,8,9^, leaving our understanding of ICL2 hampered by the lack of structural, functional and mechanistic insight.

Herein, using X-ray crystallography, we report the first structures of ICL2. Enzymatic assays and molecular dynamics calculations reveal that ICL2 is activated by the binding of acetyl-CoA or propionyl-CoA. Our results provide strong evidence that ICL2 may act as a gate-keeping enzyme, allosterically regulating the glyoxylate shunt and the methylcitrate cycle, an essential mechanism during chronic infection when *Mtb* uses lipids as the primary carbon source.

## Results

### Structure of ligand-free ICL2

**Fig. 1:**
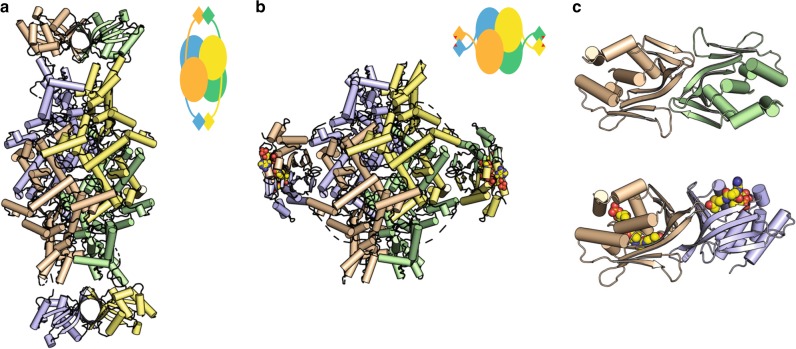
Structure of ICL2. **a** Ligand-free ICL2 forms an elongated structure with C-terminal dimers at each end of the structure. **b** Striking structural rearrangement of ICL2 upon binding to acetyl-CoA. In both **a** and **b**, each monomer is shown in different colours and the schematics outline the structural features of the tetramer in each case. **c** The dimeric association of the C-terminal domains in the ligand-free (top) and acetyl-CoA-bound (bottom) ICL2. Acetyl-CoA is shown as spheres in panels **b** and **c**. Both panels are shown with the same orientation of the wheat-coloured monomer

We determined the crystal structure of ligand-free ICL2 from *Mtb* CDC1551 at 1.8 Å resolution (Fig. 1a). The 766-residue ICL2 monomer crystallised as a tetramer, with four subunits forming an elongated structure (length ~200 Å). Each subunit comprises two distinct domains, with the N-terminal (residues 1–590) and the C-terminal (residues 603–766) domains connected by a flexible linker (residues 591–602) (Supplementary Fig. 3). The N-terminal domain contains the α/β-barrel core common to all ICLs and is packed similarly to that in *Mtb* ICL1^9^. It also possesses an active site loop containing the conserved catalytic motif ^213^KKCGH^217^, as in ICL1. However, the ICL2 N-terminal domain possesses an additional helical substructure that is not present in *Mtb* ICL1 (helices α10–α16; residues 278–427) (Supplementary Fig. 3). While such a structural insert is uncommon in bacterial ICLs, it is often present in fungal homologues^10,11^.

The C-terminal domain of *Mtb* ICL2 is unique to this isoform, with no sequence homology to known proteins. However, structural searches^12–14^ revealed similarities to members of the Gcn5-related *N*-acetyltransferase (GNAT) superfamily, despite showing only 5–15% sequence identity (Supplementary Fig. 4). The C-terminal domains from two monomers associate at each end of the ICL2 structure (Fig. 1c), forming a barrel-like structure. The association appears weak, as confirmed by small-angle X-ray scattering (SAXS), which gives an experimental scattering profile that can only be explained on the assumption that multiple conformations are present in solution (Supplementary Fig. 5). We assume that crystal packing has selected one of several accessible conformational states.

### ICL2 is activated by acetyl-CoA and propionyl-CoA

**Fig. 2:**
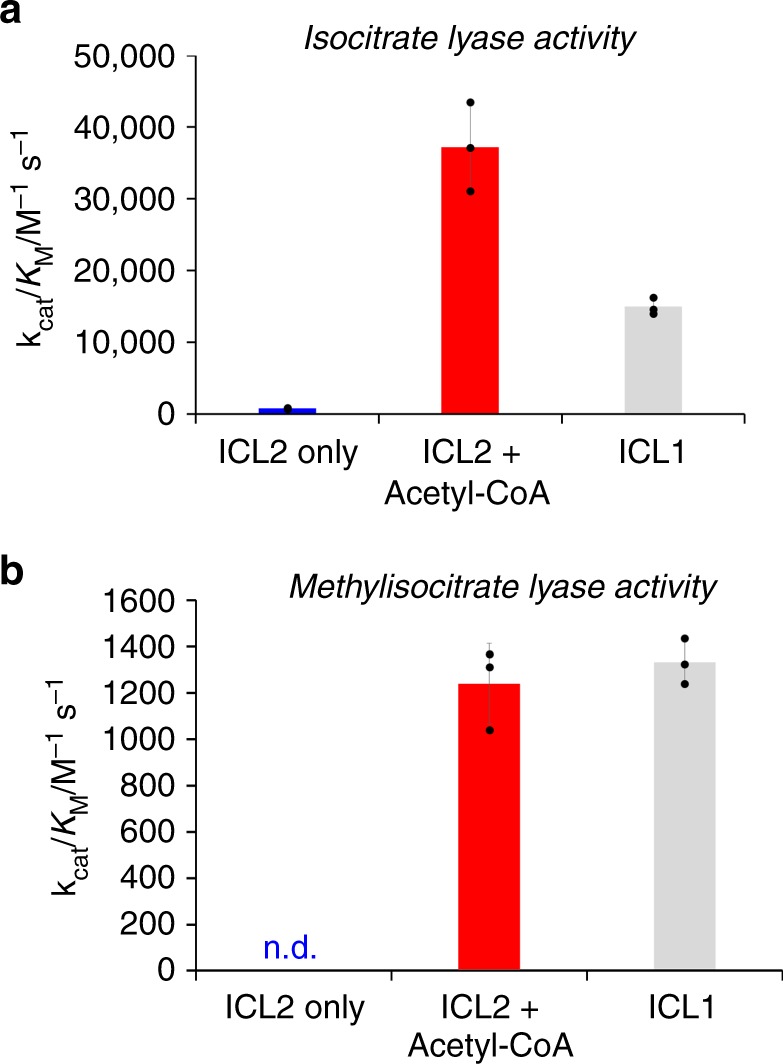
The catalytic activity of *M. tuberculosis* ICL2 is modulated by acetyl-CoA. **a** The catalytic efficiency of ICL2 is increased ~50-fold upon addition of acetyl-CoA with DL-isocitrate as a substrate. Reactions in the presence of acetyl-CoA were conducted with 0.2 μM ICL2, 100 μM–1 mM DL-isocitrate, 25 μM acetyl-CoA, 5 mM MgCl_2_ in 50 mM Tris-D11 pH 7.5 in 90% H_2_O and 10% D_2_O. Reactions in the absence of acetyl-CoA were conducted with 2 μM ICL2, 250 μM–2 mM DL-isocitrate, 5 mM MgCl_2_ in 50 mM Tris-D11 pH 7.5 in 90% H_2_O and 10% D_2_O. Reaction temperature was 27 °C. The uncorrected concentrations of the substrate DL-isocitrate were used. The error bars indicate standard deviations for three independent experiments. *K*_M_ and *k*_cat_ values of ICL1 were obtained from ref. ^15^. **b** While no detectable level of methylisocitrate turnover was observed with ICL2 only, acetyl-CoA also increased the catalytic efficiency of ICL2 when methylisocitrate was used as a substrate. The *k*_cat_/*K*_M_ value in the presence of acetyl-CoA is comparable to the value obtained for ICL1. 2-Methylisocitrate was synthesised according to literature^15^. ICL2 reactions were conducted with 1 μM ICL2, 250 μM–2 mM 2-methylisocitrate, 25 μM acetyl-CoA, 5 mM MgCl_2_ in 50 mM Tris-D11 pH 7.5 in 90% H_2_O and 10% D_2_O. ICL1 reactions were conducted with 2 μM ICL1, 250 μM–2 mM 2-methylisocitrate, 5 mM MgCl_2_ in 50 mM Tris-D11 pH 7.5 in 90% H_2_O and 10% D_2_O. Reaction temperature was 27 °C. The error bars indicate standard deviations for three independent experiments. Source data are provided as a Source Data file

The structural resemblance of the *Mtb* ICL2 C-terminal domain to members of the GNAT superfamily prompted us to investigate the potential modulation of ICL2 activity by acetyl-CoA, which is the main product of fatty acid β-oxidation. The activity of *Mtb* ICL2 was measured by a nuclear magnetic resonance (NMR)-based assay that directly monitors reaction turnover (Supplementary Fig. 6a)^15^ and a fluorescence-based continuous assay that relies on the reaction between glyoxylate and phenylhydrazine (Supplementary Fig. 6b)^16^. Both assays gave comparable results; in agreement with previous studies^6^, ICL2 showed poor isocitrate lyase activity using DL-Isocitrate as a substrate (*k*_cat_/*K*_M_ 755 ± 70 M^−1^ s^−1^ at 27 °C) (Fig. 2a, Supplementary Fig. 7 and Supplementary Table 1). However, in the presence of acetyl-CoA, ICL2 displayed a remarkable 50-fold increase in catalytic efficiency (*k*_cat_/*K*_M_ 37,200 ± 6000 M^−1^ s^−1^ at 27 °C) (Fig. 2a, Supplementary Fig. 7 and Supplementary Table 1). The *K*_M_ value for the cofactor acetyl-CoA is 2.9 ± 0.5 μM (Supplementary Fig. 8), indicating it is a relatively strong binder to *Mtb* ICL2. The presence of the inactive L-isomer of isocitrate did not affect the catalytic activity of *Mtb* ICL2 or its allosteric activation by acetyl-CoA (Supplementary Fig. 10). This increase in catalytic activity upon the addition of acetyl-CoA appeared to be specific to *Mtb* ICL2, as addition of acetyl-CoA to *Mtb* ICL1 has no effect on the catalytic activity (Supplementary Fig. 11).

We then tested the methylisocitrate lyase activity of *Mtb* ICL2; no methylisocitrate lyase activity was detected with *Mtb* ICL2 alone (Fig. 2b, Supplementary Fig. 7 and Supplementary Table 1). However, *Mtb* ICL2 was found to catalyse the turnover of methylisocitrate as a substrate in the presence of acetyl-CoA to an extent similar to ICL1 (*k*_cat_/*K*_M_ 1240 ± 175 M^−1^ s^−1^ and 1330 ± 100 for ICL2 and ICL1, respectively) (Fig. 2b and Supplementary Table 1). Propionyl-CoA, an intermediate of odd-chain fatty acid oxidation and cholesterol metabolism, also leads to an increase in the isocitrate lyase and methylisocitrate lyase activity of ICL2 (Supplementary Figs. 7, 8 and 9). Succinyl-CoA and CoA also activate ICL2, albeit to a lesser extent (Supplementary Fig. 7). Overall, our results imply that ICL2 occupies an unprecedented role in the glyoxylate shunt and methylcitrate cycle of *Mtb*.

### Acetyl-CoA triggers striking conformational changes in ICL2

To understand these results, we determined the crystal structure of *Mtb* ICL2 in the presence of acetyl-CoA at 2.36 Å resolution (Fig. 1b). One molecule of acetyl-CoA binds to each of the C-terminal domains in the tetramer, inducing striking conformational changes. Acetyl-CoA binding is mediated by extensive hydrogen bond interactions, which are exclusively formed with residues in the C-terminal domains (Supplementary Fig. 12). In the acetyl-CoA-bound structure, the C-terminal domain from one monomer moves an average of 77 Å towards the centre of ICL2 and rotates ~176° to form a new dimer with the C-terminal domain from the opposing monomer (Fig. 1c). The two C-terminal dimers formed when acetyl-CoA binds seem to be more stable as indicated by the buried interface area (1105 Å^2^) compared with the area in the ligand-free form (975 Å^2^). These distinctive conformational changes seen in the crystal structures are confirmed by the SAXS scattering profiles for ICL2 in the presence of acetyl-CoA, which are markedly different from that of ligand-free ICL2 (Supplementary Fig. 5).

Molecular dynamics (MD) simulations for both the ligand-free and acetyl-CoA-bound ICL2 structures were conducted to investigate the mechanism of the observed allosteric activation of *Mtb* ICL2 upon acetyl-CoA binding. Both structures show similar overall residue flexibilities, with local changes in regions associated with the reorganisation of the C-terminal domains (Supplementary Fig. 13a). Upon binding of acetyl-CoA, the loop consisting of residues 637–643 becomes more rigid due to the formation of the new dimer interface between the C-terminal domains. On the other hand, residues 382–388 become slightly more flexible, due to loss of interactions with the C-terminal domains resulting from the conformational change. Comparing the average structures of the C-terminal domains obtained from MD simulations shows two regions with clear local conformational changes, including residues 635–640 and residues 730–739 (Supplementary Fig. 13b). Both regions are involved in the formation of the new dimer interface between C-terminal domains upon acetyl-CoA binding (Supplementary Fig. 13c). Comparison of the C-terminal domain dimers by superimposition at one monomer shows a rotation between the monomers upon acetyl-CoA binding (Supplementary Fig. 13d). Such rotation requires a displacement of ~26 Å at the start of the C-terminal domain (residue 603). However, due to the constraints from the length of the linker region, such large movements would be prohibited in the ligand-free conformation, making the repositioning of the C-terminal domains necessary in order to form the new dimers upon acetyl-CoA binding.

**Fig. 3:**
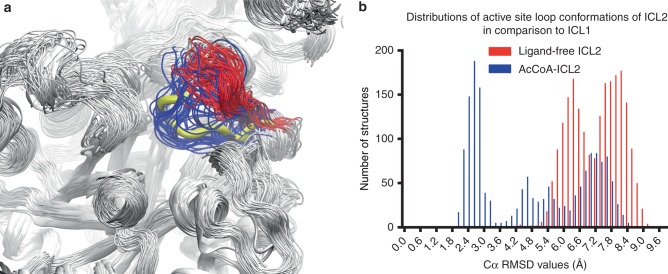
Active site loop conformations in ICL2. **a** Active site loop (residues 213–217) conformations sampled during MD simulation for ligand-free (red) and acetyl-CoA-bound (blue) ICL2. The active site loop in crystal structure of ICL1 in complex with isocitrate (PDB 3P0X) is superimposed and displayed as thick yellow ribbons. The rest of the enzyme structures are displayed in white for clarity. **b** Distributions of the RMSD values between the active site loop in ICL1 (residues 183–187, PDB 3P0X) and those in ligand-free and acetyl-CoA-bound ICL2 (residues 213–217)

Comparison of the average conformations of the N-terminal domain (residues 1–590) obtained from MD simulations reveals that the only region with clear conformational change upon acetyl-CoA binding is in the active site loop (Supplementary Fig. 13e). The changes in the conformations of the active site loop were closely examined and compared with the conformations observed in crystal structures of isocitrate-bound ICL1 from *Brucella melitensis* (*Bm* ICL1, PDB 3P0X). Results suggest that in acetyl-CoA-bound ICL2 the conformations of the active site loop are more similar to that found in the crystal structure of substrate-bound *Bm* ICL1 (Fig. 3a). The root-mean-square deviation (RMSD) values between the active site loop in ICL1 (residues 183–187) and that in both ligand-free and acetyl-CoA-bound ICL2 (residues 213–217) were calculated using the trajectories obtained from MD simulations. The distribution of the RMSD values in acetyl-CoA-bound ICL2 suggests a shift in the active site loop conformation towards a more catalytically-relevant conformation akin to that in substrate-bound *Bm* ICL1 (Fig. 3b). This is also confirmed by replica exchange molecular dynamic simulations (REMD), which was used to examine the conformations sampled by the active site loop. Our results from the REMD simulations also indicate that the active site loop largely samples different conformations in the ligand-free and acetyl-CoA-bound states (Supplementary Fig. 14). This conformational shift likely contributes to the underpinning mechanism of the observed activation of ICL2 by acetyl-CoA.

The correlated residue motions during MD simulations reveal two regions with high correlation, which interestingly corresponds to the ICL2-unique helical substructure in the N-terminal domain (α10–α16, residues 278–427), and the C-terminal acetyl-CoA binding domain (Supplementary Fig. 15a). Residues involved in correlated motions with either of the two regions were identified (Supplementary Table 2), which show opposite patterns between ligand-free and acetyl-CoA-bound ICL2. In the ligand-free enzyme, N-terminal domain residues are mostly affected by the motion of helices α10–α16 (Supplementary Fig. 15b), whereas in acetyl-CoA-bound ICL2, residues from the N-terminal domain mostly move in correlation with the C-terminal domain. Two residues from the active site loop (residues 214 and 215) showed correlation with the C-terminal domain in acetyl-CoA-bound ICL2, indicating the repositioning of the C-terminal domains has an influence on the movement of the active site loop (Supplementary Fig. 15c).

## Discussion

The regulation of carbon flux between the tricarboxylic acid (TCA) cycle and glyoxylate shunt is critical for *Mtb*, especially given its ability to utilise multiple carbon sources simultaneously^1^. Recent reports have attempted to dissect the potential mechanisms that control carbon flux between the two cycles^17–20^. None of these studies, however, have addressed the potential regulatory role(s) of ICL2, whose function in *Mtb* lipid metabolism remains poorly understood. We have shown that activation of the isocitrate lyase activity of ICL2 occurs upon acetyl-CoA and propionyl-CoA binding, thus uncovering the unique role this isoform plays in the allosteric regulation of the glyoxylate shunt at high lipid concentrations. It appears that ICL2 acts as a gate-keeping enzyme to shift the carbon flux between the TCA cycle and the glyoxylate shunt, especially when lipids are utilised as the main carbon source. In addition to its role in the glyoxylate cycle, we have also unravelled the role of ICL2 in the methylcitrate cycle. Although ICL2 does not appear to have any methylisocitrate lyase activity in the absence of allosteric activators, we have shown that acetyl-CoA and propionyl-CoA switch on the methylisocitrate lyase activity of ICL2. The allosteric activation of ICL2 in response to increasing cellular propionyl-CoA may be a mechanism to alleviate the toxicity of the methylcitrate cycle intermediates during growth on odd-chain fatty acids or cholesterol^21,22^. Overall, these observations provide a molecular-level understanding of ICL2 function in *Mtb* metabolism. The structural and mechanistic details reported herein provide the basis for the development of inhibitors against both ICL isoforms as potential antitubercular agents^23^.

## Methods

### Recombinant protein production

A synthetic gene encoding ICL2 of *M. tuberculosis* CDC1551 was obtained from Integrated DNA Technologies (Supplementary Table 3), and then subcloned into pYUB28b^24^ for protein production (Supplementary Table 4). Recombinant ICL2 was expressed in *Escherichia coli* BL21 (DE3) LOBSTR cells transformed with the pGro7 plasmid (Takara Bio Inc.) expressing GroEL/GroES chaperones under the *araB* promoter. ICL2 was produced by leaky expression at 37 °C in Terrific Broth media containing 50 μg/mL each of hygromycin and chloramphenicol. L-Arabinose (final concentration 0.1%) was added to the culture media when the cell density reached an OD_600_ of between 0.4 and 0.6. This was followed by further incubation at 37 °C for 16 h. The His_6_-tagged ICL2 was purified by immobilised metal affinity chromatography (IMAC) and size exclusion chromatography (Supplementary Methods). Purified ICL2 aliquots were flash-frozen in 20 mM HEPES pH 7.5, 150 mM NaCl and 1 mM β-mercaptoethanol and kept at −80 °C until use.

### Structural search

The C-terminal domain of ICL2 (residues 595–764) was compared against the structures in the Protein Data Bank (PDB) using the DALI server (http://ekhidna2.biocenter.helsinki.fi/dali/)^12^ and the 3D-BLAST Protein Structural Search online server (http://3d-blast.life.nctu.edu.tw/)^13,14^.

### Enzyme kinetics using NMR spectroscopy

Enzyme kinetics were measured using an NMR-based method developed in our laboratory^15^. In brief, all NMR experiments were conducted at a ^1^H frequency of 500 MHz using a Bruker Avance III HD spectrometer equipped with a BBFO probe. The instrument was operated by TopSpin 3.1 software. Experiments were conducted at 300 K. The pulse tip-angle calibration using the single-pulse nutation method (Bruker “pulsecal” routine) was undertaken for each sample. Water suppression was achieved using the excitation sculpting method. Unless otherwise stated, the number of transients was 16, and the relaxation delay was 2 s. Reactions were initiated by the addition of *Mtb* ICL2. The lag time between the addition of enzyme and the end of the first experiment was usually 4 min. All measurements were performed in triplicate. All experiments were conducted in 50 mM Tris-D11 (pH 7.5) in 90% H_2_O and 10% D_2_O (500 μL volume). Five millimolar MgCl_2_ is also added to the reaction mixture. The exact components and their concentrations for each experiment are described in the respective figure and table legends. Initial rates were obtained by non-linear curve fitting using SigmaPlot 13.0 with the “Single, 2 Parameter equation under Exponential Rise to Maximum” equation category (*f* = *a*(1 − e^−*bx*^)), in which *f* denotes the concentration of the product succinate and *x* denotes time.

### Enzyme kinetics using phenylhydrazine-coupled UV/vis assay

Enzyme kinetics were measured as described^16,25^. In brief, all experiments were conducted using transparent Nuclon Delta Surface 96-well plates (Thermo Scientific) with a Perkin Elmer EnSpire Multimode Reader operating at room temperature (~21 °C). All experiments were conducted in 50 mM Tris (pH 7.5) in 100% H_2_O (100 μL volume). Five millimolar MgCl_2_ and 10 mM phenylhydrazine are also added to the reaction mixture. Reactions were initiated by the addition of *Mtb* ICL2 into the assay mixtures. The enzymatic product glyoxylate reacted with phenylhydrazine to form a phenylhydrazone adduct (*ε*_324_ = 17,000 M^−1^ cm^−1^), which was measured spectrophotometrically at 324 nm. The absorbance was converted to concentration using a calibration curve that was constructed using known concentrations of glyoxylate and phenylhydrazine in the same reaction buffer. Initial rates were obtained by using linear regression line fitting in regions where turnover of the substrate was <10%. All measurements were performed in triplicate. The exact components and their concentrations for each experiment are described in the respective figure and table legends.

### Protein crystallography

Ligand-free ICL2 crystals were obtained by sitting drop vapour diffusion, using a protein solution comprising 8 mg/mL ICL2, 1 mM succinate and 1 mM MgCl_2_ with the Morpheus screen^26^. Cubic-shaped crystals typically grew in ~15 days using a precipitant comprising 9% w/v PEG 4000, 18% v/v glycerol, an amino acid mix (0.02 M each of L-glutamate, DL-alanine, glycine, DL-lysine, DL-serine) and 0.1 M MES/imidazole pH 6.9. Diffraction data were collected using the MX1 and MX2 beamlines at the Australian Synchrotron. All datasets were indexed and processed using XDS^27^, and scaled with AIMLESS^28^ from the CCP4 programme suite^29^. Full data collection and processing statistics are given in Supplementary Table 5.

The structure was solved by molecular replacement with Phaser^30^ using isocitrate lyase of *Aspergillus nidulans* (PDB 1DQU)^10^ as a search model. This resulted in a protein model that contained only the N-terminal domain. The initial structure was improved to obtain the complete protein model containing both N- and C-terminal domains by visual inspection and model building with cycles of automatic (phenix.autobuild^31^) and manual (COOT^32^) model building. This structure was refined at 2.90 Å resolution using REFMAC5^33^.

Additive screens around the most promising condition using the Morpheus additive screen, resulted in a crystal that formed in the presence of 0.001 M sodium tungstate dihydrate. A new dataset was collected from this crystal and used to solve the structure, using the afore-mentioned ligand-free ICL2 as a search model. The final structure was refined to 1.8 Å resolution, with crystallographic *R*_value_ of 16.83% and *R*_free_ of 20.25%. Water molecules were identified by their spherical electron density and appropriate hydrogen bond geometry with the surrounding structure. Despite the presence of succinate in the crystallisation drops, no clear electron density could be observed for this molecule. Full refinement statistics are shown in Supplementary Table 6.

Co-crystals of an ICL2-acetyl-CoA complex were grown from a solution containing 6 mg/mL ICL2, 1 mM acetyl-CoA and 1 mM MgCl_2_ (crystal form I). The best diffracting crystals were obtained in a solution comprising 8% w/v PEG 4000, 16% v/v glycerol, amino acids mix (0.02 M each of L-glutamate, DL-alanine, glycine, DL-lysine, DL-serine) and 0.1 M MES/imidazole pH 6.5. The structure was solved by molecular replacement with MOLREP^34^ using the ligand-free ICL2 structure as a search model. An initial partial model was obtained using just the N-terminal domain of ligand-free ICL2, followed by using this partial solution as a fixed model to search for the C-terminal domain. Model building using phenix.autobuild and COOT resulted in a complete model that was refined at 2.67 Å resolution, with crystallographic *R*_value_ of 21.87% and *R*_free_ of 24.10%. The structure showed unambiguous electron density for acetyl-coA in all four subunits, with no clear electron density for succinate.

In an additional experiment, 3-nitropropionic acid (3-NP), an inhibitor of ICL1^35^, was also added to the co-crystallisation drops containing ICL2, acetyl-CoA and MgCl_2_ (crystal form II). A crystal was obtained from a solution containing of 8% w/v PEG4K, 16% v/v glycerol, alcohols mix (0.02 M each of 1,6-hexanediol, 1-butanol, (*RS*)-1,2-propanediol, 2-propanol, 1,4-butanediol, 1,3-propanediol) and 0.1 M MES/imidazole pH 6.9, which diffracted to 2.36 Å resolution, and was solved using the form I structure as a search model. This structure, with crystallographic *R*_value_ of 19.82% and *R*_free_ of 23.24%, showed unambiguous electron density for acetyl-coA in all four subunits, albeit with no clear electron density for either succinate or 3-NP. Full refinement statistics are shown in Supplementary Table 6. Given the higher resolution of the form II structure, we have used this to discuss the ICL2-acetyl-CoA complex throughout the paper. The PDB_redo program^36^ was used in the final stages of refinement for all structures. All structural figures in the paper are produced using Pymol^37^.

### Small-angle X-ray scattering (SAXS) analyses

ICL2 protein aliquots were extensively dialysed against 20 mM HEPES pH 7.5, 150 mM NaCl, 5% glycerol (v/v), and 1 mM TCEP (tris(2-carboxyethyl)phosphine). This buffer was used to make the ligand solutions and to dilute protein samples. The 96-well plates containing the samples were mounted on a temperature-controlled mount at 283 K for autosampling and capillary flow data acquisition, comprising consecutive 1-s X-ray exposures. SAXS data were collected on the Australian Synchrotron SAXS/WAXS beamline and processed using the scatterBrain software package^38^. FoXS and MultiFoXS servers^39^ were used to compute SAXS profiles of ICL2 structures (X-ray structure as well as calculated population-weighted ensembles) to the experimental profiles (Supplementary Table 7).

### Molecular dynamics simulations

MD simulations were conducted using NAMD 2.12^40^ and trajectories were visualised and analysed in VMD^41^. Crystal structures for ligand-free and acetyl-CoA-bound ICL2 obtained in this study were used as starting points for MD simulations. Initial force field topology and parameters for acetyl-CoA were obtained from CGenFF server (https://cgenff.paramchem.org)^42–45^, from which parameters were assigned by analogy to existing parameters in the force field. A penalty score is given to each assigned parameter and those with large penalty values were further refined by Force Field Toolkit^46^. Additional explicit TIP3 water molecules were added to solvate the protein molecules in a water box in VMD. Na^+^ and Cl^−^ ions were added to balance the net charge of the water box. MD simulations were conducted with CHARMM36 force field^47^ at a constant temperature and pressure (298 K, 1 atm). The cutoff distance for van der Waals interactions was set to 12 Å. In each simulation, the system was first minimised for 5000 steps followed by dynamics simulation conducted with 2 fs time steps. Three MD simulations were conducted for each of ligand-free and acetyl-CoA-bound *Mtb* ICL2, initiated with different random seeds. MD simulations were conducted for 274.5, 277 and 276.9 ns for ligand-free enzyme, and 232.4, 236.6 and 236 ns for acetyl-CoA-bound *Mtb* ICL2. The total simulation time for ligand-free enzyme is 828.4 ns and that for the acetyl-CoA-bound enzyme is 705 ns. Considering there are four identical chains on the symmetrical homotetramer of *Mtb* ICL2 in each simulation, effectively the total amount of simulation time for each protein chain is 3.3 and 2.8 μs for ligand-free and acetyl-CoA-bound *Mtb* ICL2, respectively. Trajectory frames were collected every 100 ps. All MD simulations for ligand-free and acetyl-CoA-bound ICL2 fully equilibrated after 100 ns of simulations, as indicated by the protein backbone RMSD values (Supplementary Fig. 16). All analyses of MD trajectories were conducted using the collected frames from the equilibrated time period. The correlations between residue fluctuations during MD simulations were analysed. The fluctuations were obtained by calculating the RMSD values for each residue inclusive of the side chain atoms, between each frame of trajectory. The correlation coefficients between the residue fluctuations were then computed as defined by the Pearson product-moment correlation coefficient, in equation

1 $$R_{ij} = \frac{{C_{ij}}}{{\sqrt {C_{ii} \times C_{jj}} }},$$

where $C_{ij}$ is the covariance of $x_i$ and $x_j$; $C_{ii}$ and $C_{jj}$ are the variance of $x_i$ and $x_j$, respectively. The calculations and analyses of the correlation matrices were conducted using python v3.6.5 and Jupyter Notebook v5.5.0.

### Replica exchange molecular dynamics (REMD)

REMD simulations (parallel tempering) were conducted using NAMD 2.12^40^ and trajectories were visualised and analysed in VMD^41^. The same starting points for ligand-free and acetyl-CoA-bound ICL2 as in the MD simulations were used. Two sets of REMD were conducted for each of the ligand-free and acetyl-CoA-bound ICL2 states. The first set is set up with 10 replicas exchanging between a lower-temperature range of 300–310 K, and the second set contains 10 replicas exchanging at a higher-temperature range of 310–320 K. Attempts to exchange were made every 1000 steps of simulation. For the lower temperature REMD set, 51.2 ns of trajectories per replica were collected for ligand-free system, and 48.8 ns per replica collected for acetyl-CoA-bound system. For the high temperature REMD, 23.3 ns per replica were collected for the ligand-free system and 22.7 ns per replica were collected for the acetyl-CoA-bound system.

### Reporting summary

Further information on research design is available in the Nature Research Reporting Summary linked to this article.

## Supplementary information

Supplementary Information

Peer Review File

Reporting Summary

Source Data

## Data Availability

The coordinates and structure factors have been deposited in the Protein Data Bank under accession codes 6EDW, 6EDZ and 6EE1. The source data underlying Fig. 2a and b and Supplementary Figs. 6a, b, 7a, b, 8a, b, c, d, 9a, b, c, d, 10 and 11 are provided as a Source Data file. Other data are available from the corresponding authors upon reasonable request.
