# Supplementary material for: Acetyl-CoA-mediated activation of Mycobacterium tuberculosis isocitrate lyase 2
Source: Nat Commun. 2019 Oct 11;10:4639. doi: 10.1038/s41467-019-12614-7 (PMC6788997; doi:10.1038/s41467-019-12614-7)
Supplement: Supplementary file 1 — Supplementary Information [file 41467_2019_12614_MOESM1_ESM.pdf]

SUPPLEMENTARY INFORMATION

Acetyl-CoA-mediated activation of *Mycobacterium tuberculosis* isocitrate lyase 2

Ram Prasad Bhusal *et al.*

Supplementary Figures

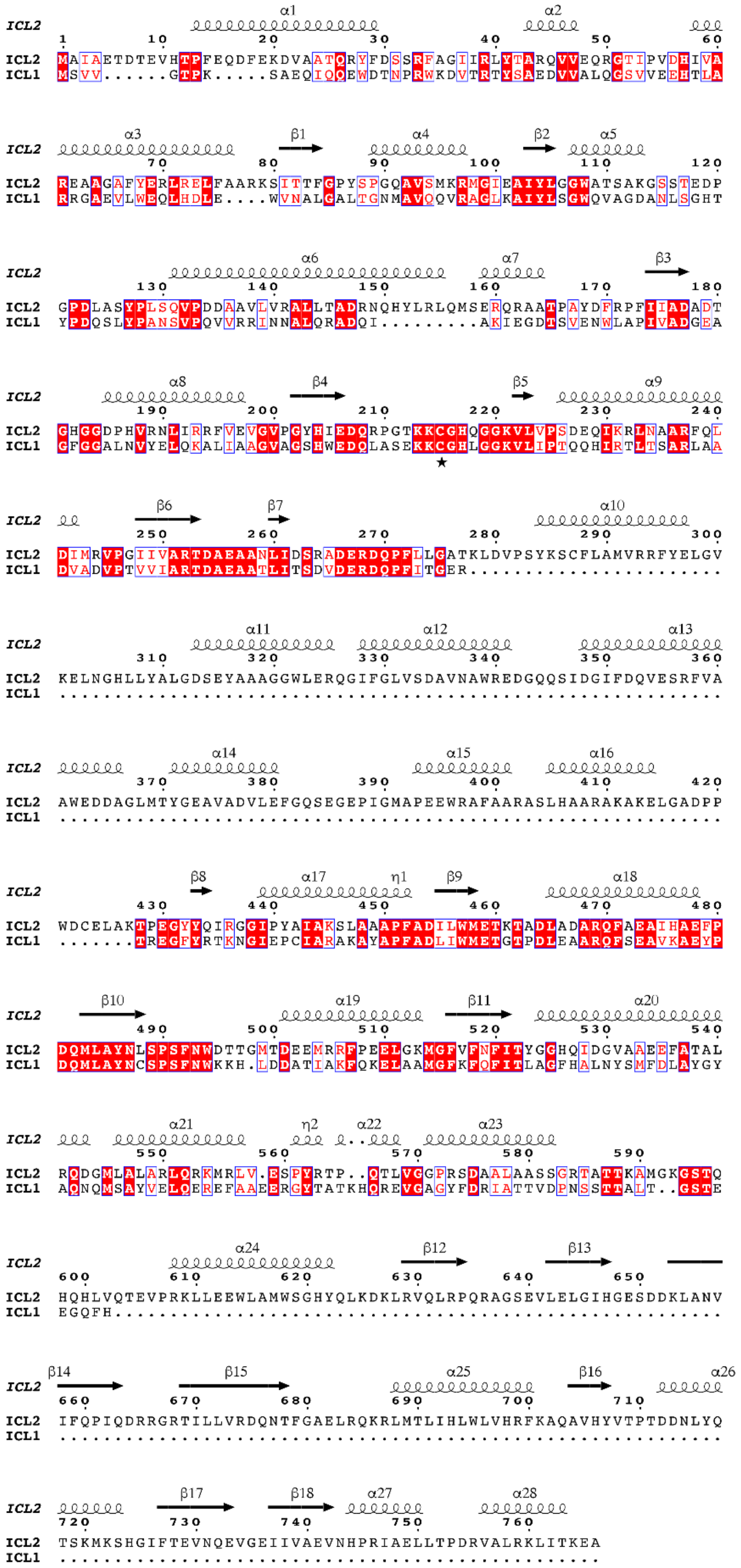

Supplementary Figure 1 | Sequence alignment of *M. tuberculosis* ICL1 and ICL2. Pairwise sequence alignment of *M. tuberculosis* ICL1 and ICL2 isoforms. Secondary structure elements for ICL2 are shown above the sequences. ICL2 contains two large inserts (residues 278–427 and residues 603–766) compared to ICL1. Both ICL1 and ICL2 contain the catalytic motif KKCGH, with the catalytic residue (Cys215) indicated by an asterisk. The figure is generated using ESpript[1].

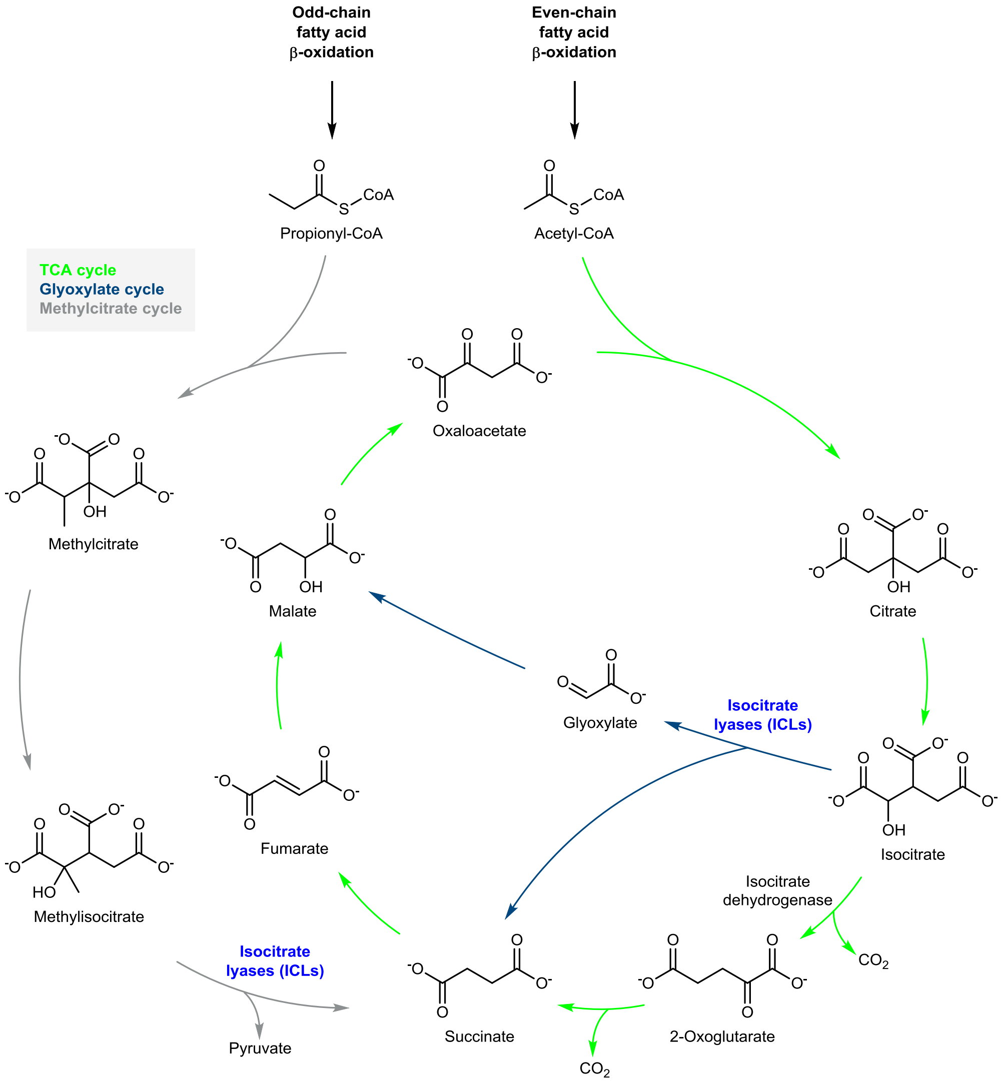

Supplementary Figure 2 | Overview of the TCA, glyoxylate and methylcitrate cycles in *M. tuberculosis*. The figure shows the roles of ICLs in *M. tuberculosis* lipid metabolism. ICLs compete with isocitrate dehydrogenase for isocitrate at the branch point of the TCA and glyoxylate cycles, bypassing two decarboxylation steps in the TCA cycle. ICLs also act as a methylisocitrate lyase in the methylcitrate cycle, catalysing the conversion of methylisocitrate into pyruvate and succinate.

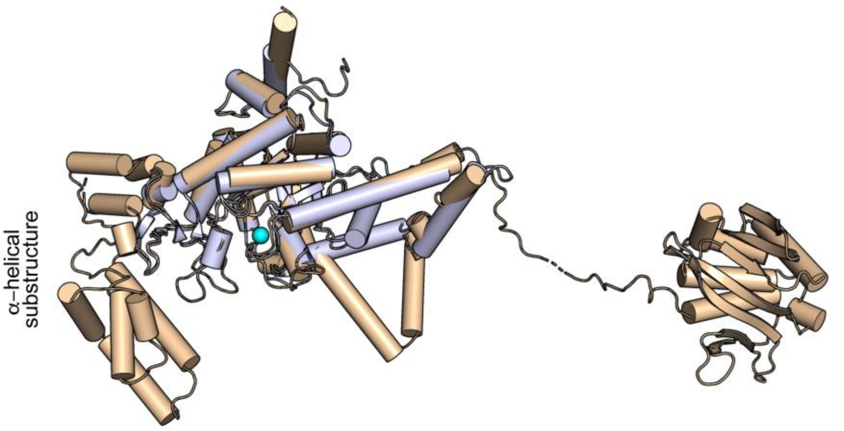

Supplementary Figure 3 | Superimposed structures of the ICL1 and ICL2 monomers. Both proteins are shown as cartoon models, with ligand-free ICL1 (PDB code 1F61) in light blue and ligand-free ICL2 in wheat color. The N-terminal domain of ICL2 has a core fold similar to that of ICL1 (root-mean-square difference of 1.28 Å over 389 aligned Cα atoms), with an additional helical substructure that is not present in ICL1. The cyan sphere represents Mg^{2+} in the ICL1 active site. The C-terminal domain, specific to ICL2, is separated from the N-terminal domain by a long and flexible linker. All structural figures in this manuscript are produced with PyMOL (Schrödinger, New York, USA).

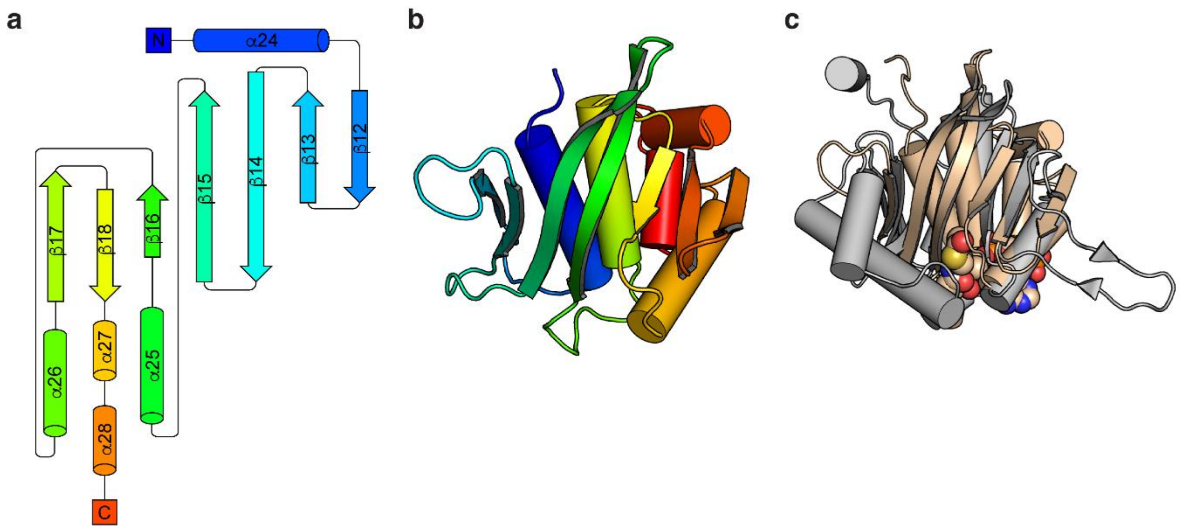

Supplementary Figure 4 | Structure of *M. tuberculosis* ICL2 C-terminal domain. (a) Topology diagram[2] for the ICL2 C-terminal domain, shown in rainbow colors, blue to red, from N- to C-terminus. Secondary structures are labelled based on the full-length ICL2 structure. **(b)** Cartoon representation of the ICL2 C-terminal domain colored as panel (a). **(c)** Structure of the ICL2 C-terminal domain (wheat) superimposed on the top hit from the DALI search[3] (histone H4- and H2A-specific amino-terminal acetylation NatD of *Schizosaccharomyces pombe*, PDB code 4UA3) (gray, *Z* score 10.4, 10% sequence identity, root-mean-square difference 3.0 Å over 123 aligned Cα atoms). The ICL2 C-terminal domain does not contain helices equivalent to the two α-helices proposed to comprise the binding site for the acceptor substrate in members of the GNAT superfamily (two gray helices at the front). The acetyl-CoA bound to the ICL2 C-terminal domain is shown in CPK form.

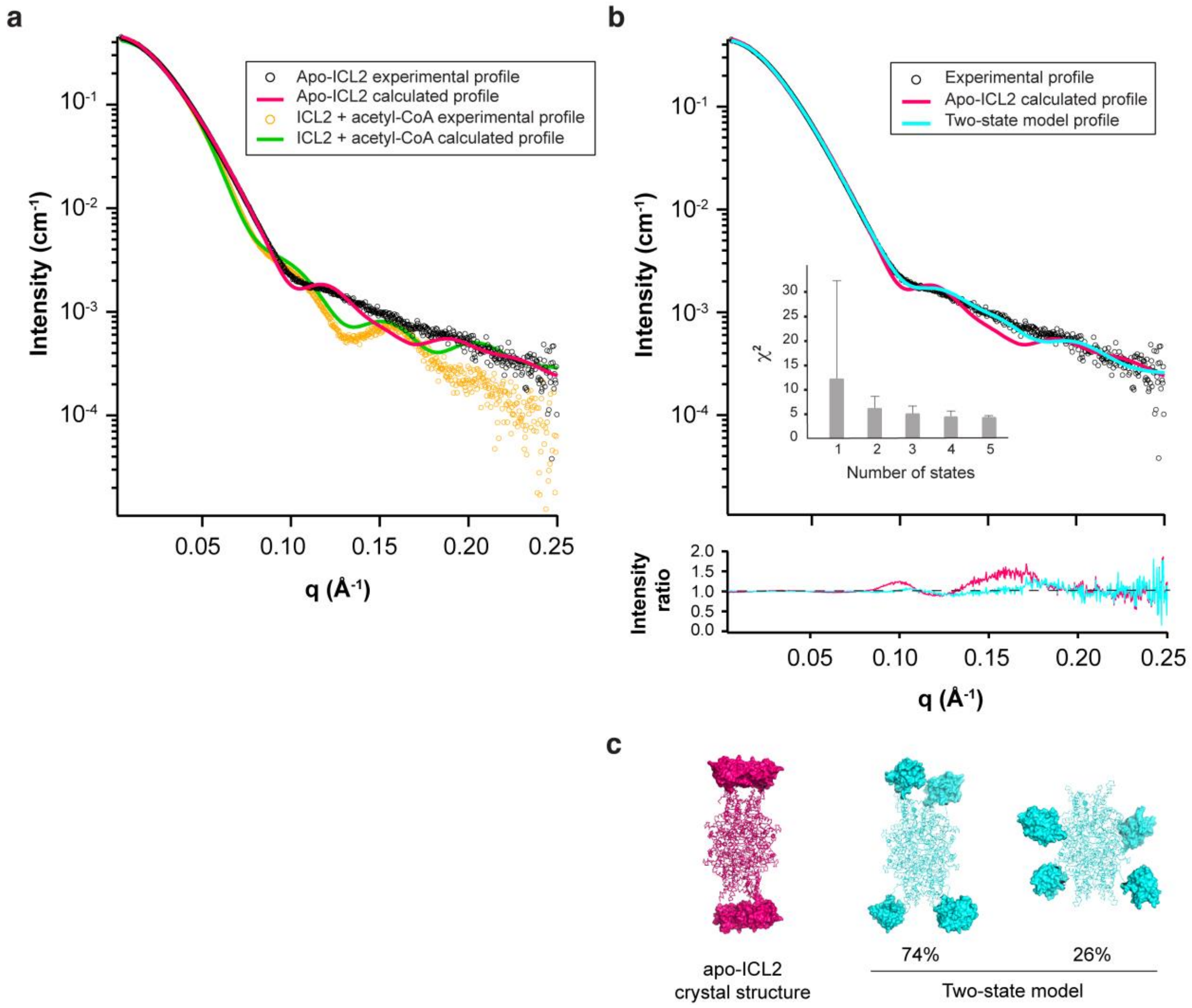

Supplementary Figure 5 | Small-angle X-ray scattering analyses of *M. tuberculosis* ICL2. (a) Small-angle X-ray scattering (SAXS) profile of ICL2 in the ligand-free state (black) and in the presence of acetyl-CoA (orange), indicating a considerable difference in ICL2 conformation in these two states. Calculated scattering profiles are also shown for the X-ray structures of ligand-free ICL2 (red) and ICL2-acetyl-CoA complex (green). **(b)** The ligand-free ICL2 crystal structure did not show a good fit to the experimental SAXS profile (red profile, χ^2=61.53), as calculated by FoXS[4]. The MultiFoXS server was then used to sample over 10,000 conformations and calculate multi-state models, resulting in significantly improved χ^2 values for single-state (χ^2=12.46) and two-state (cyan profile, χ^2=6.27) models (inset). **(c)** Variation between the X-ray structure of ligand-free ICL2 (red) and the calculated two-state model (cyan), with the corresponding weighting of each state. The C-terminal domains are shown as surface representation for easier comparison of different states.

a

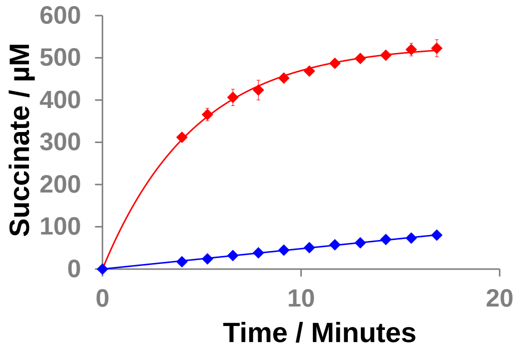

b

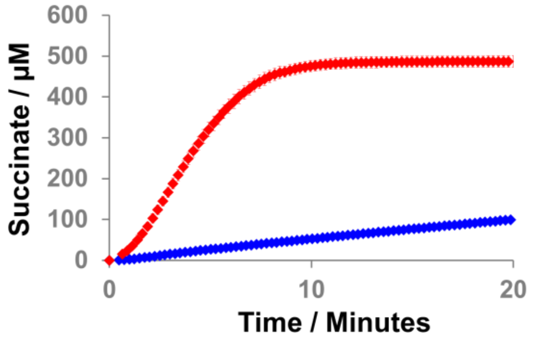

Supplementary Figure 6 | Comparison of reaction time course of *M. tuberculosis* ICL2 with DL-isocitrate as substrate by NMR assay and by UV/Vis assay. (a) NMR assay: reaction time course of ICL2-catalysed turnover of DL-isocitrate to succinate and glyoxylate. Reactions were conducted with ICL2 on its own (blue) and ICL2 + acetyl-CoA (red). Reactions were conducted with 0.5 µM ICL2, 1 mM DL-isocitrate, 1 mM acetyl-CoA/propionyl-CoA (where applicable), 5 mM $MgCl_2$ in 50 mM Tris-D11 pH 7.5 in 90% H_2O and 10% D_2O. Reaction temperature was 27 °C. The uncorrected concentrations of the substrate DL-isocitrate were used. The error bars indicate standard deviations for 3 independent experiments. **(b)** UV/Vis assay: reaction time course of ICL2-catalysed turnover of DL-isocitrate to succinate and glyoxylate. Reactions were conducted with ICL2 on its own (blue) and ICL2 + acetyl-CoA (red). Reactions were conducted with 0.5 µM ICL2, 1 mM DL-isocitrate, 25 µM acetyl-CoA (where applicable), 5 mM $MgCl_2$, 10 mM phenylhydrazine in 50 mM Tris pH 7.5 in 100% H_2O. Reaction temperature was room temperature (~21 °C). The error bars indicate standard deviations for 3 independent experiments. Source data are provided as a Source Data file.

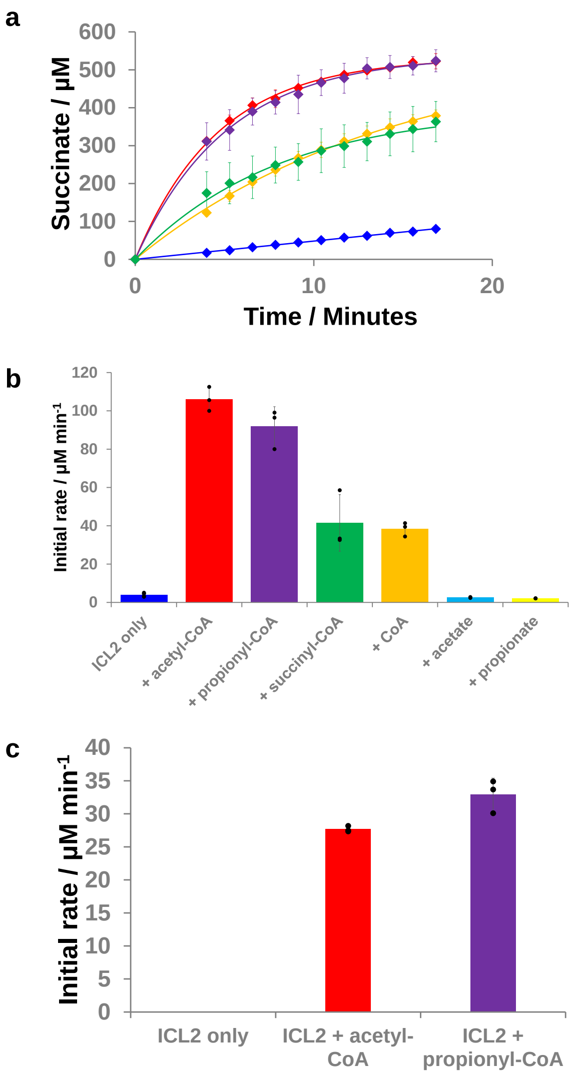

Supplementary Figure 7 | The catalytic activity of *M. tuberculosis* ICL2 is modulated by acetyl-CoA and analogues. (a) Reaction time course of ICL2-catalysed turnover of DL-isocitrate to succinate and glyoxylate. Reactions were conducted with ICL2 on its own (blue, initial rate = 3.95 ± 1.0 $\mu M\ min^{-1}$), ICL2 + acetyl-CoA (red, initial rate = 106.0 ± 6.3 $\mu M\ min^{-1}$), ICL2 + propionyl-CoA (purple, initial rate = 91.9 ± 10 $\mu M\ min^{-1}$), ICL2 + succinyl-CoA (green, initial rate = 41.5 ± 15 $\mu M\ min^{-1}$) and ICL2 + CoA (orange, initial rate = 38.4 ± 3.6 $\mu M\ min^{-1}$). Reactions were conducted with 0.5 μM ICL2, 1 mM DL-isocitrate, 1 mM acetyl-CoA/propionyl-CoA/succinyl-CoA/CoA (where applicable), 5 mM $MgCl_2$ in 50 mM Tris-D11 pH 7.5 in 90% H_2O and 10% D_2O. Reaction temperature was 27 °C. The uncorrected concentrations of the substrate DL-isocitrate were used. The error bars indicate standard deviations for 3–6 independent experiments. **(b)** Unlike acetyl-CoA (red), propionyl-CoA (purple), succinyl-CoA (green) or CoA (orange), acetate (light blue) and propionate (yellow) do not stimulate the ICL-catalysed turnover of DL-isocitrate to succinate and glyoxylate (c.f. ICL2 only, blue). Reactions were conducted

with 0.5 μM ICL2, 1 mM DL-isocitrate, 1 mM acetyl-CoA/propionyl-CoA/CoA/propionate/acetate (where applicable), 5 mM $MgCl_2$ in 50 mM Tris-D11 pH 7.5 in 90% H_2O and 10% D_2O. Reaction temperature was 27 °C. The uncorrected concentrations of the substrate DL-isocitrate were used. The error bars indicate standard deviations for 3–6 independent experiments. **(c)** Acetyl-CoA and propionyl-CoA also stimulate the methylisocitrate activity of ICL2. Reactions were conducted with 0.5 μM ICL2, 1 mM 2-methylisocitrate (substrate), 200 μM acetyl-CoA/propionyl-CoA (where applicable). 5 mM $MgCl_2$ in 50 mM Tris-D11 pH 7.5 in 90% H_2O and 10% D_2O. Reaction temperature was 27 °C. The error bars indicate standard deviations for 3 independent experiments. 2-Methylisocitrate was synthesised according to literature[5]. Source data are provided as a Source Data file.

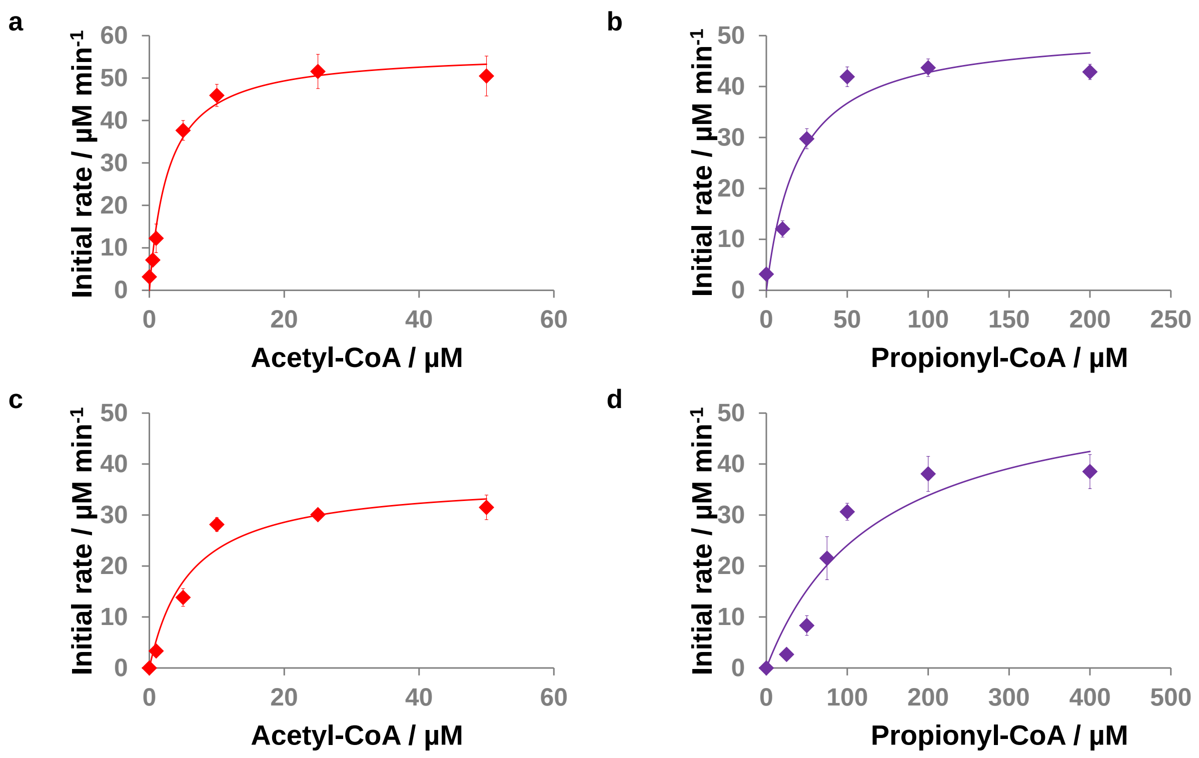

Supplementary Figure 8 | K_M measurements for cofactors acetyl-CoA and propionyl-CoA by NMR assay. (a) The K_M value for cofactor acetyl-CoA when DL-isocitrate was used as substrate was found to be 2.9 ± 0.5 µM; **(b)** The K_M value for cofactor propionyl-CoA when DL-isocitrate was used as substrate was 6.0 ± 1.1 µM; **(c)** The K_M value for cofactor acetyl-CoA when methylisocitrate was used as substrate was found to be 18.2 ± 1.8 µM; **(d)** The K_M value for cofactor propionyl-CoA when methylisocitrate was used as substrate was found to be 68.0 ± 11.8 µM. The data in (d) appeared to be sigmoidal. This could be due to poor signal-to-noise as the rate of methylisocitrate turnover was slow at low propionyl-CoA concentrations. Further studies were therefore performed by the UV/vis assay (see Supplementary Figure 9), which confirmed that the data is not sigmoidal. Reactions with DL-isocitrate were conducted with 0.2 µM ICL2, 1 mM DL-isocitrate (substrate), and varying concentration of acetyl/propionyl-CoA, 5 mM $MgCl_2$ in 50 mM Tris-D11 pH 7.5 in 90% H_2O and 10% D_2O. Reaction temperature was 27 °C. Reactions with methylisocitrate were conducted with 1 µM ICL2, 1 mM 2-methylisocitrate (substrate), and varying concentrations of acetyl/propionyl-CoA, 5 mM $MgCl_2$ in 50 mM Tris-D11 pH 7.5 in 90% H_2O and 10% D_2O. Reaction temperature was 27 °C. The error bars in the four graphs indicate standard deviations from three independent experiments. Source data are provided as a Source Data file.

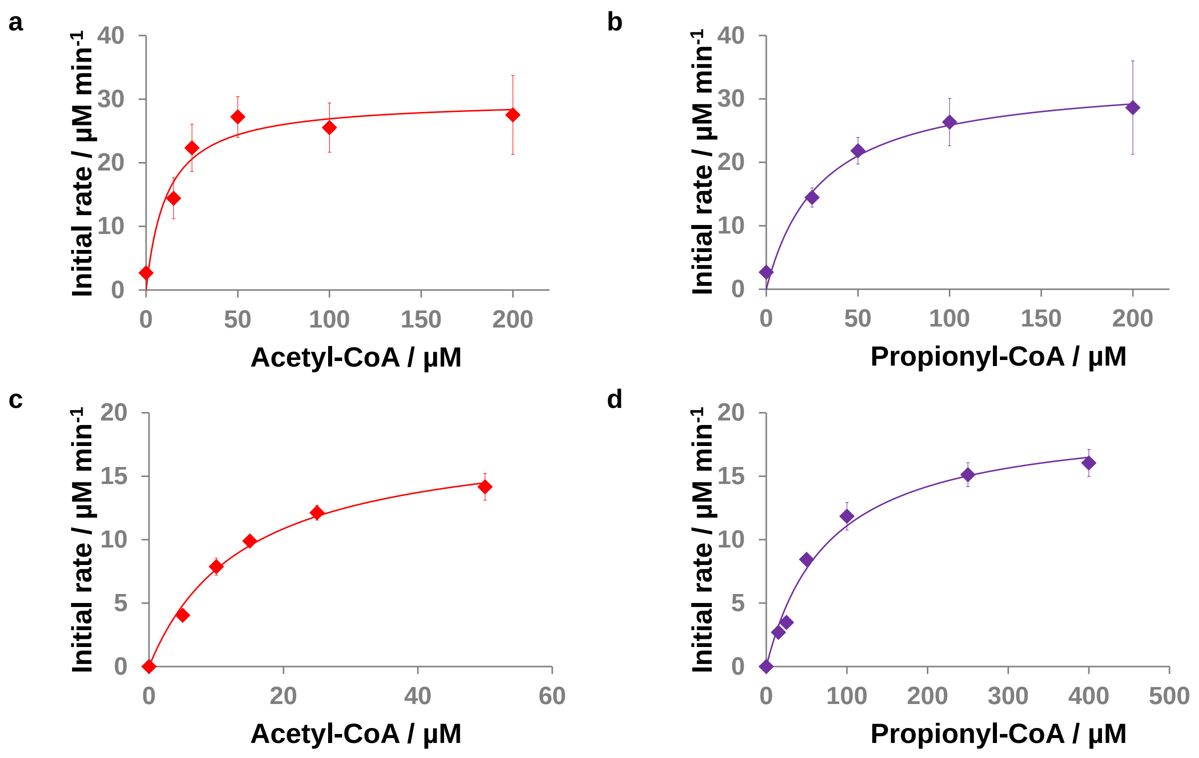

Supplementary Figure 9 | K_M measurements for cofactors acetyl-CoA and propionyl-CoA by UV/vis assay. (a) The K_M value for cofactor acetyl-CoA when DL-isocitrate was used as substrate was found to be 13.5 ± 10 μM; **(b)** The K_M value for cofactor propionyl-CoA when DL-isocitrate was used as substrate was 31.9 ± 14 μM; **(c)** The K_M value for cofactor acetyl-CoA when methylisocitrate was used as substrate was found to be 14.3 ± 1.1 μM; **(d)** The K_M value for cofactor propionyl-CoA when methylisocitrate was used as substrate was found to be 77.3 ± 2.0 μM. Reactions with DL-isocitrate were conducted with 0.2 μM ICL2, 1 mM DL-isocitrate (substrate), and varying concentration of acetyl/propionyl-CoA, 5 mM $MgCl_2$ in 50 mM Tris-D11 pH 7.5 in 90% H_2O and 10% D_2O. Reaction temperature was 27 °C. Reactions with methylisocitrate were conducted with 1 μM ICL2, 1 mM 2-methylisocitrate (substrate), and varying concentrations of acetyl/propionyl-CoA, 5 mM $MgCl_2$ in 50 mM Tris pH 7.5 in 100% H_2O. Reaction temperature was room temperature (~21 °C). The error bars in the four graphs indicate standard deviations from three independent experiments. Source data are provided as a Source Data file.

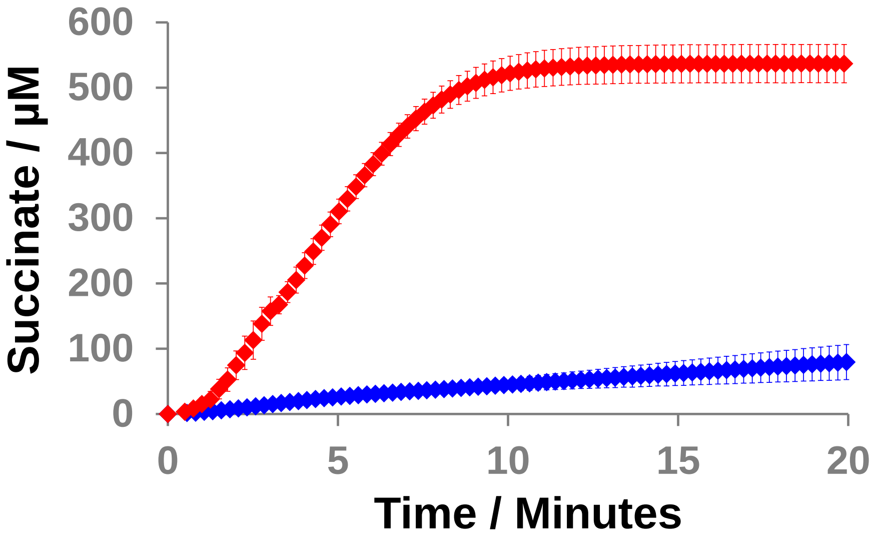

Supplementary Figure 10 | Reaction time course of *M. tuberculosis* ICL2 with D-isocitrate as substrate as monitored by the phenylhydrazine-coupled UV/Vis assay. Reaction time course of ICL2-catalysed turnover of D-isocitrate to succinate and glyoxylate. Reactions were conducted with ICL2 on its own (blue) and ICL2 + acetyl-CoA (red). Reactions were conducted with 0.2 µM ICL2, 500 µM D-isocitrate, 25 µM acetyl-CoA (where applicable), 5 mM $MgCl_2$, 10 mM phenylhydrazine in 50 mM Tris pH 7.5 in 100% H_2O. Reaction temperature was room temperature (~21 °C). The error bars indicate standard deviations for 3 independent experiments. Source data are provided as a Source Data file.

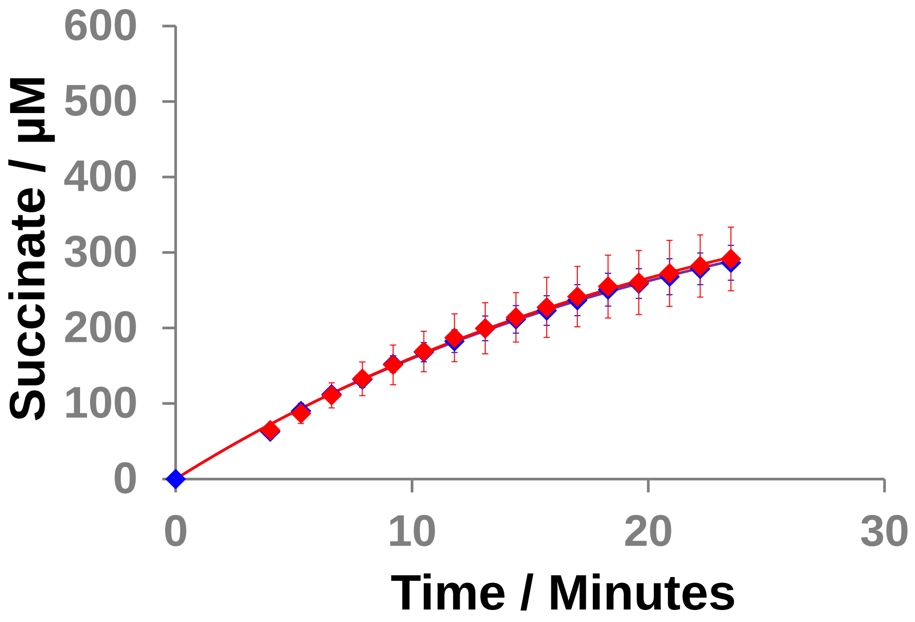

Supplementary Figure 11 | Reaction time course of *M. tuberculosis* ICL1 with DL-isocitrate as substrate as monitored by NMR assay. Reaction time course of ICL1-catalysed turnover of DL-isocitrate to succinate and glyoxylate. Reactions were conducted with ICL1 on its own (blue) and ICL1 + acetyl-CoA (red). Reactions were conducted with 190 nM ICL1, 1 mM DL-isocitrate, 5 mM acetyl-CoA (where applicable), 5 mM $MgCl_2$ in 50 mM Tris-D11 pH 7.5 in 90% H_2O and 10% D_2O. The uncorrected concentrations of the substrate DL-isocitrate were used. The error bars indicate standard deviations for 3 independent experiments. Source data are provided as a Source Data file.

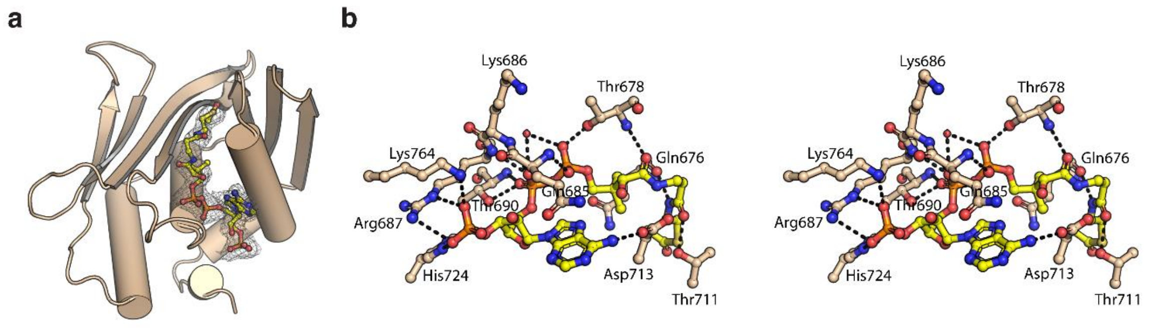

Supplementary Figure 12 | Acetyl-CoA binding to the ICL2 C-domain. (a) Cartoon representation of the ICL2 C-terminal domain with bound acetyl-CoA shown in $2F_o$ - F_c omit density (generated by Phenix[6]), contoured at 2.0 σ, and drawn as a ball-and-stick model. **(b)** Stereo-view of the acetyl-CoA binding site, with acetyl-CoA (yellow carbon atoms) and protein side chains (wheat carbon atoms) represented in ball-and-stick mode. The pyrophosphate moiety of acetyl-CoA interacts with a water molecule, shown as a red sphere, rather than with an Mg^{2+} ion, as observed in members of the GNAT superfamily. As water molecules and Mg^{2+} have virtually the same electron density, the identity of the water molecule was confirmed by 1 ps molecular dynamics calculations. The water molecule was also defined as Mg^{2+} and parallel dynamics calculations run for comparison. The water molecule was stable within the crystal structure forming hydrogen bonds whereas the ion was clearly pushed out of the ensemble. Hydrogen bond contacts are shown as dashed lines.

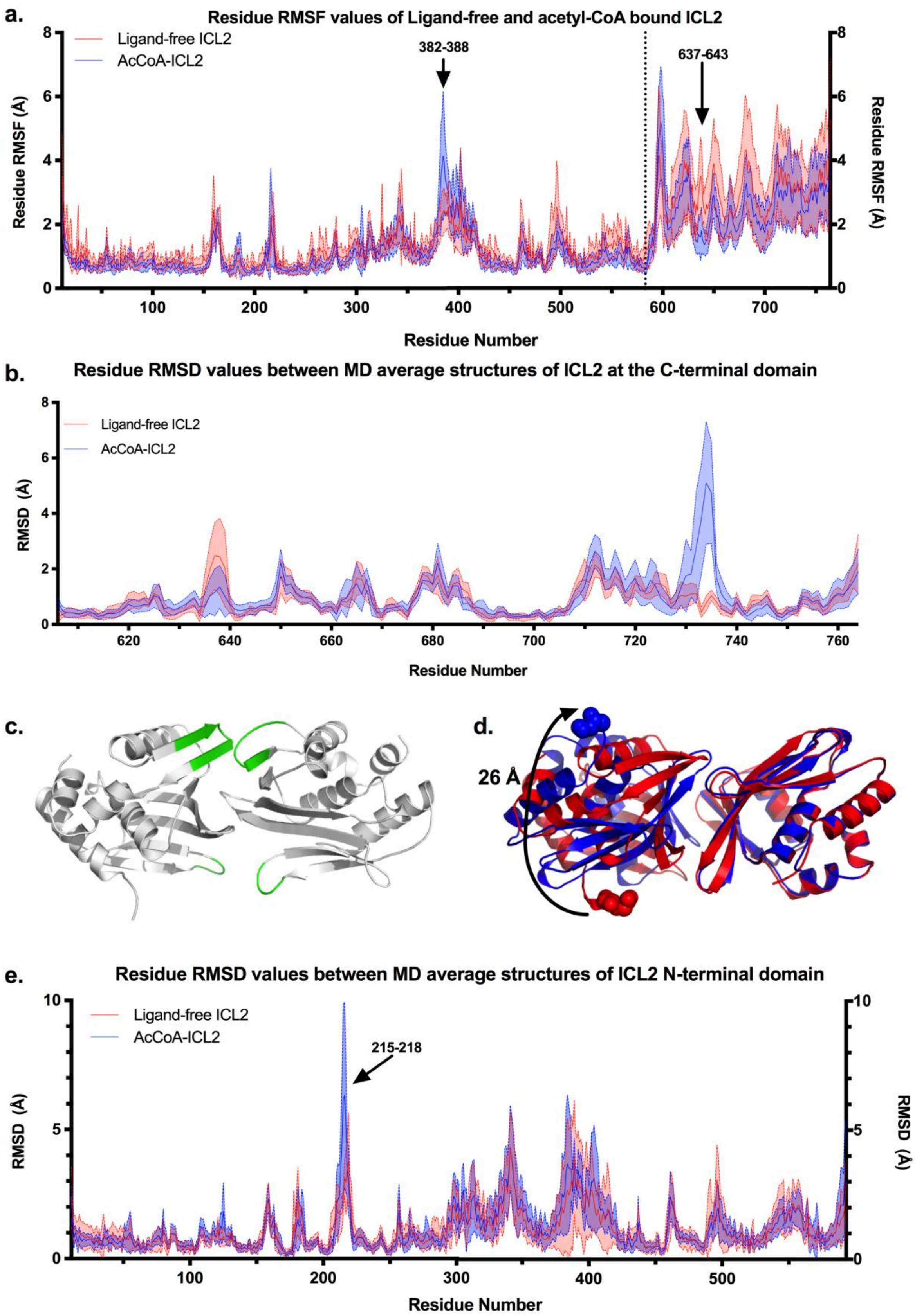

a.
Residue RMSF values of Ligand-free and acetyl-CoA bound ICL2
Ligand-free ICL2
AcCoA-ICL2
382-388
637-643
Residue RMSF (Å)
Residue Number
b.
Residue RMSD values between MD average structures of ICL2 at the C-terminal domain
Ligand-free ICL2
AcCoA-ICL2
RMSD (Å)
Residue Number
c.
d.
26 Å
e.
Residue RMSD values between MD average structures of ICL2 N-terminal domain
Ligand-free ICL2
AcCoA-ICL2
215-218
RMSD (Å)
Residue Number

Supplementary Figure 13 | Residue flexibilities and average conformations of ligand-free and acetyl-CoA-bound ICL2. (a) Chain averaged residue RMSF values of ligand-free (red) and acetyl-CoA bound (blue) ICL2 during MD simulations. Black dashed lines indicate the starting position of the C-terminal domain linker (residue 583). **(b)** Comparison of MD average conformations of the C-terminal domain (606-764) with chain A of the crystal structure of ligand-free ICL2 as the reference. (c) Regions with altered average conformations in the C-terminal domain mapped onto crystal structure of acetyl-CoA bound ICL2 (green). **(d)** Superimposition of MD average structure of C-terminal dimer at one chain to illustrate the direction of the rotation between the two C-terminal domains, in order to form the new dimer interface upon acetyl-CoA binding. Acetyl-CoA-bound ICL2 C-terminal domain is shown in blue and that of ligand-free ICL2 is shown in red. The first residues of the C-terminal domain (residue 603) were shown as spheres. **(e)** Comparison of MD average conformations of the N-terminal domain (1-593) with chain A of the crystal structure of ligand-free ICL2 as the reference. In **(a),** (**b**) and **(e)**, the average values are shown by solid lines, and error bars associated with the average values are represented by dashed lines both above and below the average data point. Areas between the error bars are colour shaded.

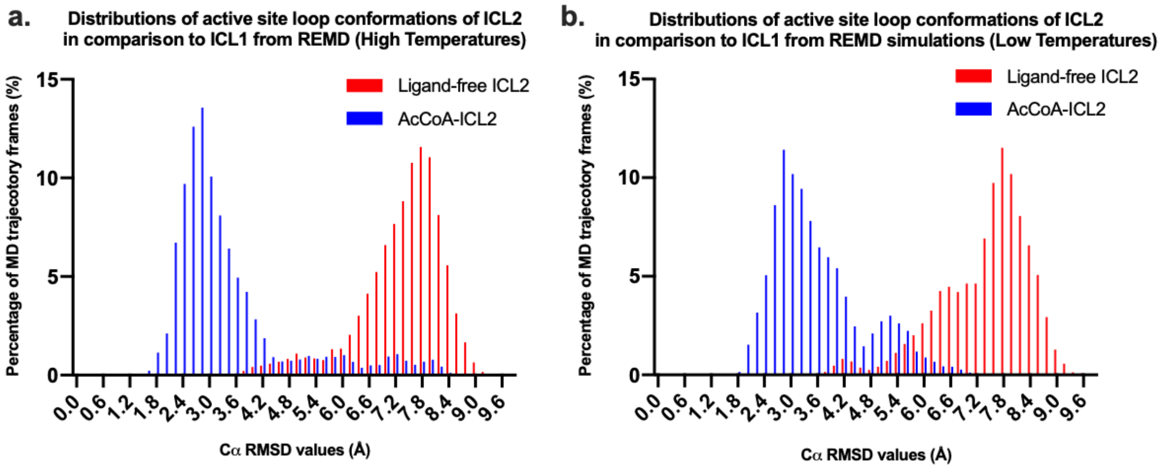

Supplementary Figure 14 | Active site loop conformations in ICL2 sampled in REMD calculations. Percentage distributions of the RMSD values between the active site loop in ICL1 (residues 183-187, PDB 3P0X) and those in ligand-free and acetyl-CoA-bound ICL2 (residue 213-217), sampled in (a) high temperature range REMD (310K-320K) and (b) low temperature range REMD (300K-310K).

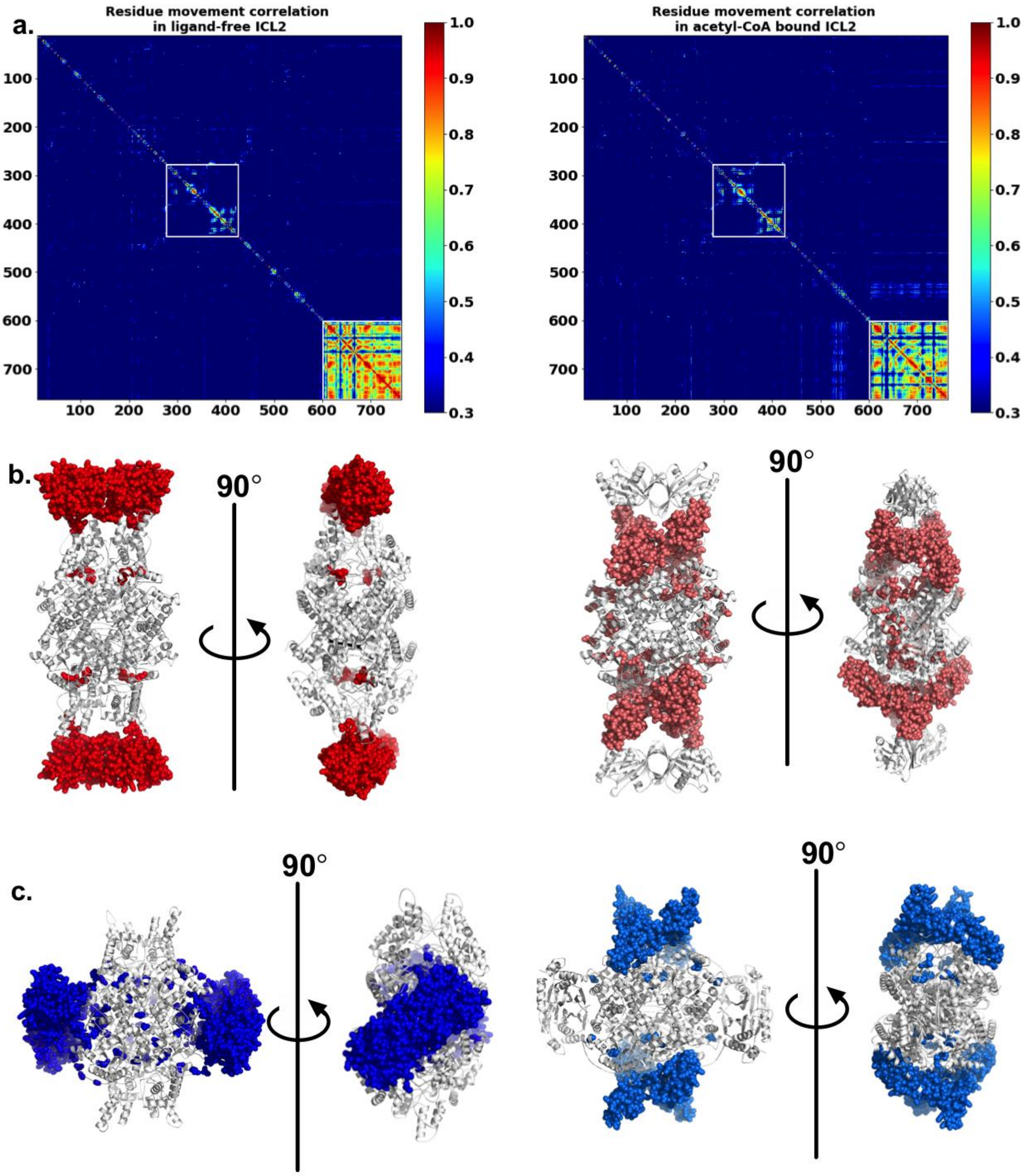

Supplementary Figure 15 | Correlations of residue fluctuations for ICL2 (a) Residue correlation matrices calculated for ligand-free and acetyl-CoA-bound ICL2. Two regions with high correlations are highlighted by white boxes. **(b)** Residues in ligand-free ICL2 that display correlative motions (correlation coefficient above 0.5) with the C-terminal domains (left, red spheres) and ICL2-unique helices (right, pink spheres). **(c)** Residues in acetyl-CoA-bound ICL2 that display correlative motions (correlation coefficient above 0.5) with the C-terminal domain (left, blue spheres) and ICL2-unique helices (right, light blue spheres).

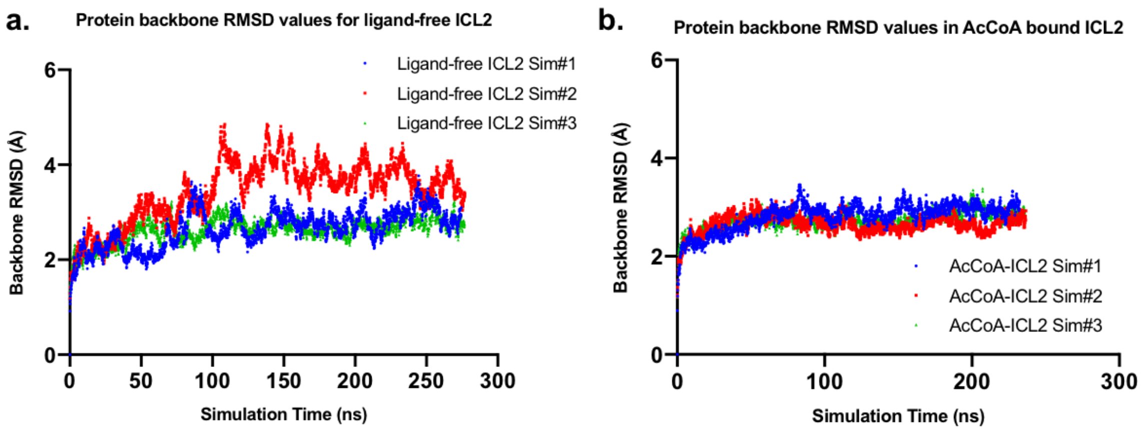

Supplementary Figure 16 | Plots of protein backbone RMSD values in the three MD simulations of the **(a)** ligand-free and **(b)** acetyl-CoA-bound ICL2.

**Isocitrate lyase activity (measured by NMR at 27 °C)**				
	**K_M of DL-isocitrate / μM**	**V_{max} / μM min^{-1}**	**k_{cat} / s^{-1}**	**k_{cat}/K_M / M^{-1} S^{-1}**
ICL2 only	606 ± 96	54 ± 3.9	0.45 ± 0.1	755 ± 68
ICL2 + acetyl-CoA	125 ± 27	55 ± 3.2	4.6 ± 0.3	37220 ± 6220
ICL1	289 ± 22	49 ± 0.1	4.3 ± 0.1	14960 ± 1190
**Methylisocitrate lyase activity (measured by NMR at 27 °C)**				
	**K_M of 2-methylisocitrate / μM**	**V_{max} / μM min^{-1}**	**k_{cat} / s^{-1}**	**k_{cat}/K_M / M^{-1} S^{-1}**
ICL2 only	No reaction	No reaction	No reaction	No reaction
ICL2 + acetyl-CoA	717 ± 215	52 ± 7.3	0.86 ± 0.1	1240 ± 175
ICL1	345 ± 39	55 ± 2.4	0.46 ± 0.1	1330 ± 97
**Isocitrate lyase activity (measured by UV/vis at room temperature ~21 °C)**				
	**K_M of DL-isocitrate / μM**	**V_{max} / μM min^{-1}**	**k_{cat} / s^{-1}**	**k_{cat}/K_M / M^{-1} S^{-1}**
ICL2 only	570 ± 130	20 ± 3.0	0.17 ± 0.1	300 ± 35
ICL2 + acetyl-CoA	250 ± 80	21 ± 8.4	1.7 ± 0.7	6940 ± 1270

Supplementary Table 1 | Kinetic analyses of the isocitrate lyase and methylisocitrate lyase activities of ICL2 in the absence and presence of acetyl-CoA. Data are means ± standard derivations from three independent experiments. Reactions by NMR spectroscopy in the presence of acetyl-CoA and DL-isocitrate as substrate were conducted with 0.2 μM ICL2, 100 μM–1 mM DL-isocitrate, 25 μM acetyl-CoA, 5 mM $MgCl_2$ in 50 mM Tris-D11 pH 7.5 in 90% H_2O and 10% D_2O. Reaction temperature was 27 °C. Reactions by NMR spectroscopy in the absence of acetyl-CoA and DL-isocitrate as substrate were conducted with 2 μM ICL2, 250 μM–2 mM DL-isocitrate, 5 mM $MgCl_2$ in 50 mM Tris-D11 pH 7.5 in 90% H_2O and 10% D_2O. Reaction temperature was 27 °C. The uncorrected

concentrations of the substrate DL-isocitrate were used. K_M and k_{cat} values of ICL1 were obtained from reference 5. Reactions by NMR spectroscopy in the presence of acetyl-CoA and 2-methylisocitrate as substrate were conducted with 1 μM ICL2, 250 μM–2 mM 2-methylisocitrate, 25 μM acetyl-CoA, 5 mM $MgCl_2$ in 50 mM Tris-D11 pH 7.5 in 90% H_2O and 10% D_2O. ICL1 reactions were conducted with 2 μM ICL1, 250 μM–2 mM 2-methylisocitrate, 5 mM $MgCl_2$ in 50 mM Tris-D11 pH 7.5 in 90% H_2O and 10% D_2O. Reactions by UV/vis spectroscopy in the presence of acetyl-CoA and DL-isocitrate as substrate were conducted with 0.2 μM ICL2, 100 μM–2 mM DL-isocitrate, 25 μM acetyl-CoA, 5 mM $MgCl_2$ and 10 mM phenylhydrazine in 50 mM Tris pH 7.5 in 100% H_2O. Reaction temperature was room temperature (~21 °C). Reactions by UV/vis spectroscopy in the absence of acetyl-CoA and DL-isocitrate as substrate were conducted with 2 μM ICL2, 250 μM–2 mM DL-isocitrate, 5 mM $MgCl_2$ in 50 mM Tris pH 7.5 in 100% H_2O. Reaction temperature was room temperature (~21 °C). The uncorrected concentrations of the substrate DL-isocitrate were used. The differences in the values between the NMR and UV/vis experiments are likely due to differences in reaction temperature.

	**Ligand-free ICL2**	**Acetyl-CoA-bound ICL2**
**Residues showing correlated motion with C-terminal domain**	207, 255, 256, 445, and 601	21, 56, 214, 215, 232, 461, 526, 528, 529, 530, 532, 533, 536, 540, 542, 543, 544, 545, 550, 556, 557, 595, and 599
**Residues showing correlated motion with ICL2-unique helices α10-α16**	72, 125, 181, 182, 188, 206, 207, 214, 270, 273, 274, 275, 276, 277, 434, 454, 459, 490, 491, 494, 528, 529, 530, 533, 550, and 594.	186, 187, 189, 264, 270, 274, 275, 276, 277, 434, 460, 461, and 504.

Supplementary Table 2 | Residues showing correlated motions (correlation coefficient above 0.5) with the N-terminal helices α10-α16 and C-terminal domain.

```
ATGGCCATCGCCGAAACGGACACCGAGGTCCACACACCGTTCGAGCAGGACTTTGAGAAAGACGTAGCCGCCACT
CAGCGATACTTCGACAGCTCGCGCTTTGCTGGGATCATTCGGCTCTACACCGCCCGCCAAGTCGTGGAACAGCGC
GGCACGATCCCCGTCGACCACATCGTGGCGCGAGAGGCGGCGGGCGCCTTCTACGAGCGTCTGCGCGAACTCTTT
GCAGCCCGCAAGAGCATCACGACGTTTGGCCCCTACTCGCCGGGGCAGGCGGTGAGCAGAAGCGGATGGGTATCG
AGGCGATCTACCTCGGTGGTTGGGCTACCTCAGCTAAGGGCTCCAGCACCGAAGATCCGGGGCCCGACCTCGCCA
GCTACCCGCTGAGCCAGGTGCCTGACGATGCCGCGGTGCTGGTGCGCGCCTTGCTCACCGCGGACCGCAACCAAC
ACTATCTACGCCTGCAGATGAGCGAGCGACAGCGTGCGGCGACACCGGCTTACGACTTCCGCCCGTTTATCATCG
CCGACGCCGACACCGGCCACGGCGGCGATCCGCACGTACGCAACCTGATCCGCCGCTTCGTCGAGGTCGGTGTGC
CGGGCTACCACATCGAGGACCAACGACCCGGCACCAAGAAGTGCGGCCACCAGGGCGGCAAGGTCCTGGTGCCGT
CCGACGAACAGATCAAGCGGCTCAACGCCGCCCGCTTCCAGCTCGACATCATGCGGGTGCCCGGCATCATCGTCG
CACGCACCGACGCGGAGGCGGCCAACCTGATCGACAGTCGCGCCGACGAGCGTGACCAGCCGTTCCTTCTCGGCG
CGACCAAGCTCGACGTACCGTCCTACAAGTCCTGTTTCCTGGCAATGGTGCGGCGTTTTTACGAACTGGGCGTCA
AGGAGCTCAATGGTCATCTTCTCTATGCGCTTGGCGACAGCGAGTACGCGGCGGCCGGCGGTTGGCTTGAGCGCC
AAGGCATTTTCGGCTTGGTCTCCGACGCGGTCAACGCGTGGCGGGAGGACGGCCAGCAGTCGATCGACGGCATTT
TCGACCAGGTCGAGTCGCGGTTCGTGGCGGCCTGGGAGGACGACGCGGGCCTGATGACCTACGGAGAGGCCGTGG
CGGACGTGCTCGAATTCGGTCAGAGCGAGGGCGAACCCATTGGCATGGCTCCCGAGGAGTGGCGGGCGTTCGCCG
CGCGTGCATCGCTGCATGCCGCCCGGGCAAAGGCCAAGGAGCTGGGCGCCGATCCGCCATGGGACTGCGAGCTGG
CCAAGACCCCGGAGGGCTACTACCAGATCCGCGGCGGCATACCGTATGCGATCGCCAAATCGCTGGCCGCGGCAC
CGTTTGCCGACATTCTTTGGATGGAGACCAAGACCGCCGATCTCGCCGACGCTCGACAGTTCGCCGAGGCGATCC
ATGCCGAGTTCCCCGACCAGATGCTGGCGTACAACCTCTCACCATCGTTCAACTGGGACACCACCGGCATGACCG
ACGAGGAGATGCGGCGCTTCCCCGAGGAGCTCGGCAAAATGGGCTTCGTCTTCAACTTCATCACCTATGGCGGGC
ACCAGATCGACGGTGTCGCGGCCGAGGAATTCGCCACCGCGCTGCGCCAGGACGGCAGCTGGCGCTGGCTCGGTT
GCAGCGCAAGATGCGCTTGGTCGAATCTCCCTATCGCACACCGCAAACGCTAGTCGGCGGGCCGCGCAGTGACGC
CGCATTGGCTGCCTCCTCCGGACGCACGGCGACCACGAAGGCAATGGGCAAGGGCTCCACCCAGCACCAGCACTT
GGTGCAAACTGAGGTGCCGCGCAAGCTGCTAGAGGAATGGCTGGCCATGTGGAGCGGTCACTACCAGCTCAAAGA
CAAACTGCGCGTACAGCTTCGGCCGCAGCGGGCCGGCTCGGAGGTGCTCGAGCTCGGCATCCACGGCGAAAGCGA
TGACAAGCTCGCCAACGTGATATTCCAACCGATCCAAGATCGCCGCGGCCGCACCATCCTGTTGGTACGCGACCA
GAACACGTTCGGTGCGGAACTACGCCAAAAGCGGCTGATGACCCTGATCCACCTCTGGCTCGTCCACCGCTTCAA
GGCGCAGGCGGTGCACTACGTCACGCCCACCGACGACAACCTCTACCAGACCTCGAAGATGAAGTCGCATGGAAT
CTTCACCGAGGTCAACCAGGAGGTGGGCGAGATCATCGTCGCCGAGGTGAACCACCCGCGCATCGCCGAACTGCT
GACGCCCGATCGGGTGGCGCTGCGGAAGTTGATCACGAAGGAGGCGTAG
```

Supplementary Table 3 | DNA sequence of ICL2. A synthetic gene (plasmid: pUCIDT) containing the sequence was obtained from Integrated DNA Technologies.

```
Forward primer

5' aactagaatcgacatATGGCCATCGCCGAAACGGACAC 3'

Reverse primer

5' aaatcaaagcttCTACGCCTCCTTCGTGATCAACTTCC 3'
```

Supplementary Table 4 | Forward and reverse primers used for subcloning. The NdeI and HindIII restriction sites were chosen for subcloning to the pYUB28b vector

	Ligand-free ICL2	ICL2-acetyl-CoA
Wavelength (Å)	0.95370	0.95372
Space group	$P2_1$	$P2_1$
Cell dimensions		
a (Å)	95.82	105.47
b (Å)	136.08	169.97
c (Å)	138.92	106.43
α, β, γ (°)	90.0, 99.59, 90.0	90.0, 95.26, 90.0
Resolution range[a] (Å)	44.09-1.80 (1.86-1.80)	48.87-2.36 (2.44-2.36)
R_{merge}[a]	0.16 (2.157)	0.157 (2.17)
R_{pim}[a]	0.087 (1.178)	0.096 (1.31)
Observed reflections[a]	2,473,507 (116,559)	1,063,582 (53,462)
Unique reflections[a]	323,023 (31,895)	152,900 (15,231)
Multiplicity[a]	7.7 (7.4)	7.2 (7.3)
Mean I/σI[a]	11.2 (1.0)	7.0 (1.0)
Completeness (%)[a]	99.84 (98.94)	99.97 (99.99)
CC(1/2)[b]	0.997 (0.39)	0.993 (0.474)

Supplementary Table 5 | Data collection and processing statistics. Values in parentheses are for the outermost resolution shell.

	Ligand-free ICL2	ICL2-acetyl-CoA
PDB code	6EDW	6EE1
Resolution range (Å)	44.09-1.80	48.87-2.36
R_{work}/R_{free} (%)	16.83/20.25	19.82/23.24
**Number of atoms (non-hydrogen)**		
Protein	23,335	22,866
Ligand	34	204
Solvent	3,486	574
**r.ms deviations from ideality**		
Bonds (Å)	0.008	0.013
Angles (°)	1.17	1.21
**Average *B* factors (Å^2)**		
Protein	30.1	65.5
Acetyl CoA	-	79.7
Glycerol	39.5	-
Mg^{2+}	21.5	-
Waters	38.6	54.8
**Ramachandran statistics (%)**		
Favored	98.97	98.39
Allowed	1.03	1.58
Outliers	0.0	0.0
Molprobity score/ percentile	2.33/99th	2.6/100th

Supplementary Table 6 | Structure refinement statistics.

Data-collection parameters		
Beamline*	AS SAXS/WAX	
Wavelength (Å)	1.0322	
Detector	Dectris – Pilatus 1M	
Camera length (mm)	3300	
q range ($Å^{-1}$)	0.006 – 0.6	
Sample capillary flow rate (ml/min)	0.5	
Total sample volume (µl)	100	
Exposure time/image (s)	1	
No. images per sample	40	
Concentration range ($mgml^{-1}$)	0.125 – 2.0	
Temperature (K)	283	
Software		
Primary data collection	*scatterBrain*	
Data processing	*scatterBrain*	
Computation of model intensities	*Crysol*	
Multi-state modeling	*MultiFoXS*	
	**Ligand-free ICL2**	**ICL2 + acetyl CoA**
Structural parameters†		
$I(0)$ (cm^{-1}) [from $P(r)$]	0.59 ± 0.0003	0.59 ± 0.0002
R_g (Å) [from $P(r)$]	52.55 ± 0.34	52.87 ± 0.34
$I(0)$ (cm^{-1}) (from Guinier)	0.59 ± 0.0004	0.59 ± 0.0003
R_g (Å) (from Guinier)	51.99 ± 0.34	51.90 ± 0.27

* Full details of the beamline specifications are available at the Australian Synchrotron website (http://archive.synchrotron.org.au/aussyncbeamlines/saxswaxs/saxs-specifications)
† For 2 $mgml^{-1}$ sample

Supplementary Table 7 | Small angle X-ray scattering parameters and statistics.

Supplementary Methods

Affinity chromatography

Affinity chromatography was conducted by using a 5 mL HisTrap HP column (GE Healthcare) connected with an ÄKTA start protein purification system (GE Healthcare). A flow rate of 1 mL/min was used. The column was charged with a charge buffer containing 50 mM $NiSO_4 \cdot 7H_2O$. It was then washed with six column volumes of binding buffer containing 20 mM HEPES, 150 mM NaCl, 20 mM imidazole and 1 mM β-mercaptoethanol (pH 7.5). After application of cell lysate, protein was eluted using elution buffer containing 20 mM HEPES, 150 mM NaCl, 500 mM imidazole and 1 mM β-mercaptoethanol (pH 7.5). All fractions with UV absorbance were analysed by SDS-PAGE. All fractions containing ICL2 were mixed and concentrated by centrifugation at 4 ºC and 3000 rpm by using a Multifuge 3 S-R benchtop centrifuge (Thermo Scientific) with Amicon Ultra-15 centrifugal filter devices (Merck Millipore) with 50 kDa MWCO.

Size exclusion chromatography

Size exclusion chromatography was conducted by using a HiPrep 16/60 Sephacryl S-100 HR column (GE Healthcare) connected with an ÄKTA start protein purification system (GE Healthcare). A flow rate of 0.5 mL/min was used. The column was equilibrated with two column volumes of buffer containing 20 mM HEPES, 150 mM NaCl and 1 mM β-mercaptoethanol (pH 7.5). The protein (concentrated to 0.5 mL) was then loaded onto the column. The protein was eluted with the same buffer in 1 mL fractions. All fractions with UV absorbance were analysed by SDS-PAGE to determine the purity of the protein. All fractions containing pure ICL2 were mixed and concentrated using Amicon Ultra-15 centrifugal filter devices (Merck Millipore) with 50 kDa MWCO. Purified ICL2 were stored at -80 ºC until use.

Supplementary References

1. Robert, X. & Gouet, P. *Nucl. Acids Res.* **42**, W320–W324 (2014).
2. Stivala, A., Wybrow, M., Wirth, A., Whisstock, J. & Stuckey, P. *Bioinformatics* **27**, 3315–3316 (2011).
3. Holm, L. & Laakso, L. M. *Nucl. Acids Res.* **44**, W351–W355 (2016).
4. Schneidman-Duhovny, D., Hammel, M., Tainer, J. A. & Sali, A. *Nucl. Acids Res* **44**, W424–W429 (2016).
5. Bhusal, R. P. *et al. Med. Chem. Commun.* **8**, 2155–2163 (2017).
6. Liebschner, D. *et al. Acta Cryst.* **D73**, 148–157 (2017).
