# Supplementary material for: Acetyl-CoA-mediated activation of Mycobacterium tuberculosis isocitrate lyase 2
Source: Nat Commun. 2019 Oct 11;10:4639. doi: 10.1038/s41467-019-12614-7 (PMC6788997; doi:10.1038/s41467-019-12614-7)
Supplement: Supplementary file 2 — Peer Review File [file 41467_2019_12614_MOESM2_ESM.pdf]

Reviewers' comments:

Reviewer #1 (Remarks to the Author):

Bhusal et al. report the in vitro activation of isocitrate lyase 2 (ICL2) from Mycobacterium tuberculosis by acetyl-CoA and propionyl-CoA. While the ICL1 isoform has been fairly well investigated, its ICL2 counterpart has received limited attention. Considering that this organism depends on fatty acids during its latent stage of infection, it is perhaps not surprising that fatty acid precursors could alter the activity of enzymes that function during this stage. The authors used kinetics and X-ray crystallography to characterize the nature of the interaction between acetyl-CoA and ICL2. Their findings are important for future studies on ICL2; however, there are substantial shortcomings in their experiments that prevents this exciting discovery from being published at this point.

The kinetics experiments were inadequately described and executed. The main text mostly describes the NMR parameters used in measurements but makes no mention of the components in solution. Supplementary Figure 6 includes some details, but only with regards to panel c (lines 62-65). Were these conditions also used in panels a and b? Also unclear is whether the authors corrected the racemic mixture of DL-isocitrate for the active D enantiomer; it is assumed that they did not. Since 1 mM was used in all cases where a concentration is mentioned, then this means that only 500 µM active substrate was present. The Keq for the reaction at pH 7.6, 27 ºC (almost identical conditions to the current study) was reported to be 0.0289 M (Smith & Gunsalus J Biol Chem 1957, 229, 305), so at equilibrium, there should only be about 100 µM succinate generated, yet the authors indicated that complete conversion had occurred in 15 min in two of the conditions. Considering that the reaction was not coupled to drive it to completion, the accuracy of the measurements is highly questionable. Additionally, the authors did not indicate how the initial rates were determined. Commonly, initial rates are measured by a linear fit to the data during the first 10% of the reaction, but with the exception of the control sample (blue), the reactions had long surpassed this point. The curves drawn through the data suggest a curve fitting procedure was applied, but no details were provided. While it is possible to obtain initial velocities from such curves, assuming they are first-order exponentials, this can contain substantial error, especially if there is a lack of data to properly define the curvature (see especially the green and yellow curves). While the authors clearly believe their 1H NMR assay is superior (ref 22), the continuous UV-vis assays are likely to be much more accurate and faster, as it is possible to monitor the first 10% conversion quite reliably.

The data in Supplementary Figure 7d look sigmoidal, suggestive of allostery. The authors should assess this with more scrutiny (after addressing the accuracy of their assay), as it would not be surprising if the binding of the CoA species influenced the activity in such a manner.

ICL1 should be tested to be sure acetyl-CoA exclusively activates ICL2 as suggested.

In the crystallization methods section, the authors attempted to crystallize ICL2 with the inhibitor 3-nitropropionate (3-NP) in the presence of acetyl-CoA and Mg2+. The authors reported that the structure of the resultant crystal "showed unambiguous electron density for acetyl-CoA in all four subunits, albeit with no clear electron density for either succinate or 3-NP." First, no mention was made that succinate was included in the crystallization mixture, so this should be amended. Second, 3-NP is actually a covalent inhibitor of ICL1, forming a thiohydroximate with the active site cysteine residue (Ray et al. ACS Chem Biol 2018, 13, 1470). Was there electron density present around this residue that could be modeled as this adduct? A molecule of pyruvate is also bound in the above complex in the glyoxylate-binding pocket, so it could be a good idea to include pyruvate or glyoxylate in the crystallization mixture with 3-NP.

An apoenzyme is an enzyme devoid of its cofactors. Because Mg2+ was still present in all structures, the use of the term "apo" is unintentionally misleading in the present context.

Why was succinate included in the crystallization mixture for "apo-ICL2"? Was it hoped that it would bind? It may be worth noting that ICL1 employs an ordered sequential mechanism, with glyoxylate being released last/bound first; thus, assuming ICL2 also follows this mechanism, it

might be best to incubate it with glyoxylate instead of succinate.

Reviewer #2 (Remarks to the Author):

Bhusal et al. have carried out an elegant study, elucidating the structure of the Mycobacterium tuberculosis isocitrate lyase 2. Unexpectedly, they report that the binding of the metabolites acetyl-CoA or propionyl-CoA to ICL2 results in an extensive structural rearrangement, increasing enzyme activity.

Overall, the manuscript is well written, and the experiments are logically presented. The joint essentiality of ICL1 and ICL2 in the virulence repertoire of M. tuberculosis was demonstrated over 13 years ago, yet the precise contribution of ICL2 remained unclear due to its low ICL and MCL activity when tested in-vitro. The results are an important step towards unravelling the enzymology of ICL2 and elucidating its precise functionality during Mtb infection. The findings are very novel and are most definitely of interest to others in the community and the wider field. Thus, I support the publication of this manuscript in Nature Communications upon addressing the comments below.
Remarks to the author:

Lines 91-96: The acetyl-CoA binding site in ICL2 was confirmed in detail with structural information (Fig S8), however, it would be nice to see some experimental verification to support the molecular dynamics simulations which propose how acetyl/propionyl-CoA binding may drive the proposed conformational shift. Site directed mutagenesis in combination with enzyme kinetics should be used to verify that residues 427-430, 358 or 359 are indeed feeding this alteration to the active site. This is particularly important as the ICL2 C-terminal domain does not contain helices equivalent to the two α-helices proposed to comprise the binding site for the acceptor substrate in members of the GNAT superfamily.

Lines 105-107: It may be also relevant to mention the ICL2 response to increasing cellular propionyl-CoA may be a mechanism to alleviate the toxicity of methylcitrate cycle intermediates during growth on fatty acids. Chemical inhibition of Mtb ICL/MCL activity with 3-NP blocks intracellular Mtb growth, which is reversible by B12 addition [1, 2]. Notably, as described in Supplementary Table 1, acetyl-CoA (or propionyl-CoA) turns on the MCL activity of ICL2. This enzyme does not appear to have any MCL activity in the absence of the allosteric activators.

1. Griffin JE, Pandey AK, Gilmore SA, Mizrahi V, McKinney JD, et al. Cholesterol catabolism by Mycobacterium tuberculosis requires transcriptional and metabolic adaptations. Chem Biol 2012;19:218–27.
2. Eoh H, Rhee KY. Methylcitrate cycle defines the bactericidal essentiality of isocitrate lyase for survival of Mycobacterium tuberculosis on fatty acids. Proc Natl Acad Sci U S A 2014;111:4976–81.

Reviewer #3 (Remarks to the Author):

The work by Bhusal et al reports the structure of Mycobacterium tuberculosis isocitrate lyase 2, which is a key enzyme in the TCA and glyoxylate cycles. Up to now, we have several structures of ICL1, However, there was no structure of ICL2 and consequently several important open questions in the field remained to be answered. By solving the ICL2 structure, this study tries to answer some of them. Interestingly, they worked out the role of Acetyl-CoA in the activation of the enzyme using both structural and functional studies. A nice combination of X-ray crystallography, biochemical assays and computational methods tries to support the paper's central concepts. I believe that the implications are of interest for the readership of Nature Communications. However, I think the current manuscript needs a major improvement in both the way of presenting the data (especially about the figures) and also certain scientific aspects of the manuscript need to be addressed more clearly, which are listed below:

1. A major point in the simulation part is about sampling. Two simulations shown in the paper are not sufficiently long and in my idea the ensemble of conformations seen in these simulations could suffer from sub-optimal sampling. Furthermore, the ensemble could also be biased by the force field and also the water model used in the study. The authors' claim that "The 84 conformations of the active site loop (residues 213-217) in acetyl-CoA-bound ICL2 displayed 85 a clear shift towards a 'closed' conformation in a similar manner to those observed in crystal 86 structures of isocitrate-bound ICL from Brucella melitensis (PDB 3P0X) and 87 nitropropionate/glyoxylate-bound Mtb-ICL1 (PDB 1F8I) (Fig. 3a and 3b), likely resulting in 88 the observed activation." would not be directly supported by the simulation data unless the further evidence demonstrates that simulations have sampled the accessible phase-space sufficiently. Therefore, a calculation probing the conformational ensemble (i.e. replica-exchange simulation, or a sufficiently long unbiased simulation where the all conformations are sampled sufficiently) is a major support for this part.
2. About the correlated motion, it is not clear how the correlated motion has been calculated. For example, it is not clear if in the calculation, only the linear correlation was considered or the non-linear correlation was also included? Therefore, it would be difficult to judge how much the correlated motions are relevant. Beside this ambiguity, based on Supp Fig 10, I could not see the correlated motion of helices α6-α10 mentioned in the manuscript (probably there are few residues with high correlation but there is no collective trend). Furthermore, There is no clear correlated motion between N-terminal and C-terminal domains (at least visually). Therefore, I do not understand the claim about the role of correlated motions in the communication of acetyl-CoA binding to the active site (Fig 3c does not help in this regard).
3. In the manuscript, the calculation of free energy of assembly dissociation has been referred to the paper by Krissinel & Henrick. However, there is no indication about the exact way the free energy has been calculated (or the equation that has been used). To make the manuscript clear and reproducible, I request to add a section in the method and describe this calculation. On the other hand, considering the error in the calculation, the $\Delta\Delta G$ of ~1.5 Kcal/mol is probably a negligible difference.
4. Another issue is the cryptic way of describing the structural search to find the similarities with the GNAT superfamily. As this part is a central part to the manuscript and the role of Acyl-CoA in the function of ICL2, it would be good also to add the exact description of the search protocol in the method section.
5. Supp Figure 11 has not been discussed in the text. However, it contains several interesting points. C-terminal domain in the apo state has a quite high RMSD, which probably means internal instability of this domain in absence of Acyl-CoA. Acetyl-CoA also has a quite high RMSD. Does such a high RMSD mean that the binding mode of Acetyl-CoA changes during the simulation? In particular between frame ~600-800 there is a sharp rise in the RMSD of Acetyl-CoA which probably requires an explanation.

Minor points
1. In general, the way of presenting figures and the coloring method could be improved.
2. Fig 3a is difficult to understand and also two Xray structures are not visible. It would be better to use transparent coloring.
3. In Supp Fig 9a and c, it is not clear how the regions with local changes were identified. For example, the region 88-92 in 9a does not seem to have high fluctuation (RMSF < 2).

Reviewer #4 (Remarks to the Author):

The major claim is that isocitrate lyase 2 of Mycobacterium tuberculosis is activated by binding acetyl-CoA. The crystal structure shows that when acetyl-CoA binds, the C-terminal domains rearrange. The core of the enzyme forms a tetramer, but the C-terminal domains dimerize. The C-terminal domains switch binding partners and interact with the tetrameric core when acetyl-CoA is bound. This is novel and will be of interest to others in the community of enzymologists and of researchers investigating M. tuberculosis. The discovery will influence thinking in the field.

I struggled to understand why the enzyme was activated on binding acetyl-CoA. Molecular dynamics simulations were used to investigate the mechanism, but it was not clear to me what

these simulations proved.

More specific comments:

Line 73 I would like to see the values for the buried surface areas, since the binding energies are inferred from these.

Lines 84-88 I don't think this observation is sufficiently described. I am not clear how Figure 3a and b show this. 3P0X is identified in the Figure, but 1F8I is not.

Line 93 These residue numbers can't be the helices - more residues are in the helices.

Line 95 Figure 3c has ICL2 unique helices written in the upper left, but it is not clear to me which they are. They are not identified in the legend.

Figure 1’s legend could be improved by explaining that the enzyme is a tetramer: The core is tetrameric, but the C-terminal domains dimerize. Although this is explained in the paper, many readers will look at the figures first.

In Figure 2, it is hard to see the error bars.

Figure 3b: I am not clear on this plot. The Figure title explains that the results from an MD simulation of ICL2 are shown. The x-axis has Calpha RMSD values - how are these so evenly spaced? Is it really a histogram? What are the number of values plotted on the y-axis? The plot is for 8 residues. Would it not be better to show RMSD values for the 8 residues? I am not clear whether the values on the left of the red plot are zero or one - but my confusion might be that I don't understand the “Number of values” plotted on the y-axis.

Figure 3c: Is the correlation in the movement of these residues?

Supplementary Figure 3: Is the additional helical substructure the part on the left? There are also two helices middle bottom in wheat. In the paper, the additional helices are alpha10-16. Perhaps labelling them here would help.

“Cartoon representation” is used where I would say ribbon diagram. I’m not sure if this terminology is from the program PYMOL.

Supplementary Figure 6 a. Why are there no points before 4 min? Are curves fitted to the points and initial rates calculated from these? Initial rates would normally be measured from the linear portion. Is the error range on the red curve correct? It seems too low. The red and purple curves do not look that different. The error for the orange curve also seems low. What is Tris-D11?

Supplementary Figure 7d does not look like a plot of Michaelis-Menten kinetics, yet there is no explanation of the S-shape.

Supplementary Figure 8. I cannot judge whether molecular dynamics calculations can confirm a water molecule. I would have thought that the coordination would distinguish water from Mg^{2+}.

Supplementary Figure 9a. I could not distinguish the plots. Am I correctly interpreting that the direction of the arrow, up or down, indicates which model shows greater flexibility in the simulation?

Supplementary Table 2. Are the data from ICL2-acetyl-CoA (form I) important? Although it is listed as a different crystal form, the space group and cell dimensions are similar to those of form II. Why not include only the better-diffracting crystal? My understanding from the protein crystallography is that the structure of the apo form was first determined at low resolution, 2.9 Å. When a crystal that diffracted to 1.8 Å was found, the model was refined against the higher resolution data set. This data set and the model are described in the tables. For the complex with

acetyl-CoA, two crystal forms are described, but other than the difference in resolution, the models seemed the same. Is there any need to discuss the first crystal form for the complex?

Reviewer #1

"The kinetics experiments were inadequately described and executed. The main text mostly describes the NMR parameters used in measurements but makes no mention of the components in solution. Supplementary Figure 6 includes some details, but only with regards to panel c (lines 62-65). Were these conditions also used in panels a and b?"

We thank Reviewer #1 for this critical insight. We have changed the experimental section in the manuscript, as well as figure and table legends in both the manuscript and supplementary information with accurate details on the components of the reaction mixtures and the concentrations that were used in each experiment.

"Also unclear is whether the authors corrected the racemic mixture of DL-isocitrate for the active D enantiomer; it is assumed that they did not. Since 1 mM was used in all cases where a concentration is mentioned, then this means that only 500 µM active substrate was present."

DL-Isocitrate was used in our studies. The concentrations of DL-isocitrate that were stated in the manuscript are based on the racemic mixture. We have updated the text, and figure and table legends in the manuscript to clarify this. In addition, we have also repeated our measurement with D-isocitrate, confirming that the presence of the L-isomer has no effect on the kinetics of *Mtb* ICL2.

"The Keq for the reaction at pH 7.6, 27 ºC (almost identical conditions to the current study) was reported to be 0.0289 M (Smith & Gunsalus J Biol Chem 1957, 229, 305), so at equilibrium, there should only be about 100 µM succinate generated, yet the authors indicated that complete conversion had occurred in 15 min in two of the conditions. Considering that the reaction was not coupled to drive it to completion, the accuracy of the measurements is highly questionable."

We thank Reviewer #1 for this very useful comment. We have studied the paper published by Smith and Gunsalus (1957) in detail and it is reported that the equilibrium lies towards the forward reaction. For example, according to Table V in the paper, when 1.67 mM isocitrate was used as substrate, almost complete turnover of the substrate was observed (1.67 mM succinate and 1.63 mM glyoxylate were formed after 30 minutes). Isocitrate was only observed (in reasonable amount) when the concentrations of succinate and glyoxylate were above 3 mM. Our results are in agreement with Smith and Gunsalus; as the effective concentration of D-isocitrate used in our study was 500 µM, complete turnover of the substrate to succinate and glyoxylate can be expected. Nonetheless, in order to confirm the accuracy of our NMR assay, we have repeated the measurements using the phenylhydrazine-coupled continuous UV/vis assay reported by Smith and Gunsalus. The UV/Vis results agree with the results that we obtained by NMR, in which complete turnover of the substrate was observed.

"Additionally, the authors did not indicate how the initial rates were determined. Commonly, initial rates are measured by a linear fit to the data during the first 10% of the reaction, but

with the exception of the control sample (blue), the reactions had long surpassed this point. The curves drawn through the data suggest a curve fitting procedure was applied, but no details were provided. While it is possible to obtain initial velocities from such curves, assuming they are first-order exponentials, this can contain substantial error, especially if there is a lack of data to properly define the curvature (see especially the green and yellow curves). While the authors clearly believe their 1H NMR assay is superior (ref 22), the continuous UV-vis assays are likely to be much more accurate and faster, as it is possible to monitor the first 10% conversion quite reliably."

We thank the Reviewer #1 for this insight. We have repeated and reanalysed our data by using non-linear curve fitting to obtain the initial rates for the NMR experiments. We have updated the experimental section in the manuscript for clarification. In addition, as suggested by the Reviewer #1, we have repeated the kinetic measurements with the phenylhydrazine-coupled continuous UV/Vis assay. Our results from the two assays are in agreement and we have updated the manuscript to include these new data.

"The data in Supplementary Figure 7d look sigmoidal, suggestive of allostery. The authors should assess this with more scrutiny (after addressing the accuracy of their assay), as it would not be surprising if the binding of the CoA species influenced the activity in such a manner."

Based on this helpful comment, we have carefully reanalysed our data. While we cannot completely rule out cooperative binding, we believe the relatively low readings at 25 µM and 50 µM propionyl-CoA are likely due to poor signal-to-noise as the rate of 2-methylisocitrate turnover was slow at low propionyl-CoA concentrations.

"ICL1 should be tested to be sure acetyl-CoA exclusively activates ICL2 as suggested."

We have repeated the experiments with ICL1 and confirmed that acetyl-CoA has no effect on ICL1. We have updated our manuscript to include this new data (Supplementary Figure 7).

"In the crystallization methods section, the authors attempted to crystallize ICL2 with the inhibitor 3-nitropropionate (3-NP) in the presence of acetyl-CoA and Mg2+. The authors reported that the structure of the resultant crystal "showed unambiguous electron density for acetyl-CoA in all four subunits, albeit with no clear electron density for either succinate or 3-NP." First, no mention was made that succinate was included in the crystallization mixture, so this should be amended. Second, 3-NP is actually a covalent inhibitor of ICL1, forming a thiohydroximate with the active site cysteine residue (Ray et al. ACS Chem Biol 2018, 13, 1470). Was there electron density present around this residue that could be modeled as this adduct? A molecule of pyruvate is also bound in the above complex in the glyoxylate-binding pocket, so it could be a good idea to include pyruvate or glyoxylate in the crystallization mixture with 3-NP."

We apologise for the confusion, as our crystallisation experiments either contained 3-NP or succinate. In both cases, crystals that were grown in the presence of either 3-NP or succinate

showed some patchy electron density in the active site that we could not confidently model and hence left it without modelling. We have tried a combination of these ligands for crystallisation and we could only obtain high-diffracting crystals in the cases that are reported in the manuscript.

"An apoenzyme is an enzyme devoid of its cofactors. Because Mg2+ was still present in all structures, the use of the term "apo" is unintentionally misleading in the present context."

We have now changed all 'apo' to 'ligand-free' in the manuscript.

"Why was succinate included in the crystallization mixture for "apo-ICL2"? Was it hoped that it would bind? It may be worth noting that ICL1 employs an ordered sequential mechanism, with glyoxylate being released last/bound first; thus, assuming ICL2 also follows this mechanism, it might be best to incubate it with glyoxylate instead of succinate."

As described above, we have used different ligands and also various combinations thereof, in the crystallisation experiments. We could only obtain high resolution diffracting crystals in the presence of succinate or succinate analogues. However, we could not confidently model succinate into the active site of our structures.

Reviewer #2

"Lines 91-96: The acetyl-CoA binding site in ICL2 was confirmed in detail with structural information (Fig S8), however, it would be nice to see some experimental verification to support the molecular dynamics simulations which propose how acetyl/propionyl-CoA binding may drive the proposed conformational shift. Site directed mutagenesis in combination with enzyme kinetics should be used to verify that residues 427-430, 358 or 359 are indeed feeding this alteration to the active site. This is particularly important as the ICL2 C-terminal domain does not contain helices equivalent to the two α-helices proposed to comprise the binding site for the acceptor substrate in members of the GNAT superfamily."

We have conducted additional MD simulations in order to increase sampling (see details in response to Reviewer #3). Due to the additional trajectories collected the correlation pathway involving residues 427-430, 358 and 359 is no longer observed. Therefore, we felt it was not necessary to make the suggested mutations. We have added a detailed analysis of the C-terminal domain dimers, in both ligand-free and acetyl-CoA bound ICL2s, to clearly show that acetyl-CoA is driving the large conformational change.

"Lines 105-107: It may be also relevant to mention the ICL2 response to increasing cellular propionyl-CoA may be a mechanism to alleviate the toxicity of methylcitrate cycle intermediates during growth on fatty acids. Chemical inhibition of Mtb ICL/MCL activity with 3-NP blocks intracellular Mtb growth, which is reversible by B12 addition [1, 2]. Notably, as

described in Supplementary Table 1, acetyl-CoA (or propionyl-CoA) turns on the MCL activity of ICL2. This enzyme does not appear to have any MCL activity in the absence of the allosteric activators.

1. Griffin JE, Pandey AK, Gilmore SA, Mizrahi V, McKinney JD, et al. Cholesterol catabolism by Mycobacterium tuberculosis requires transcriptional and metabolic adaptations. Chem Biol 2012;19:218–27.
2. Eoh H, Rhee KY. Methylcitrate cycle defines the bactericidal essentiality of isocitrate lyase for survival of Mycobacterium tuberculosis on fatty acids. Proc Natl Acad Sci U S A 2014;111:4976–81."

We thank Reviewer #2 for these excellent suggestions. We have included the extra discussion and references in our revised manuscript.

Reviewer #3

"1. A major point in the simulation part is about sampling. Two simulations shown in the paper are not sufficiently long and in my idea the ensemble of conformations seen in these simulations could suffer from sub-optimal sampling. Furthermore, the ensemble could also be biased by the force field and also the water model used in the study. The authors' claim that "The 84 conformations of the active site loop (residues 213-217) in acetyl-CoA-bound ICL2 displayed 85 a clear shift towards a 'closed' conformation in a similar manner to those observed in crystal 86 structures of isocitrate-bound ICL from Brucella melitensis (PDB 3P0X) and 87 nitropropionate/glyoxylate-bound Mtb-ICL1 (PDB 1F8I) (Fig. 3a and 3b), likely resulting in 88 the observed activation." would not be directly supported by the simulation data unless the further evidence demonstrates that simulations have sampled the accessible phase-space sufficiently. Therefore, a calculation probing the conformational ensemble (i.e. replica-exchange simulation, or a sufficiently long unbiased simulation where the all conformations are sampled sufficiently) is a major support for this part."

In order to increase sampling, we have conducted two additional MD simulations for each of the ligand-free and acetyl-CoA-bound ICL2. Each simulation was initiated with a different random seed. The final total simulation time for ligand-free ICL2 is 828.4 ns and that for acetyl-CoA-bound ICL2 is 705 ns. As there are 4 identical protein chains in each simulation, effectively we have collected 828.4 ns x4=3313.6 ns = 3.3 μs of trajectories for ligand-free ICL2, and 705 ns x4= 2820 ns=2.8 μs of trajectories for acetyl-CoA-bound ICL2. We believe the sampling problem is overcome by such a large time scale of trajectories collected, and this can be further illustrated by the broad distribution of the active site loop conformations (updated Figure 3), which suggests the system has moved out of its starting local minimum and sufficiently explored and sampled the conformational space.

"2. About the correlated motion, it is not clear how the correlated motion has been calculated. For example, it is not clear if in the calculation, only the linear correlation was considered or the non-linear correlation was also included? Therefore, it would be difficult to judge how much the correlated motions are relevant. Beside this ambiguity, based on Supp Fig 10, I could not see the correlated motion of helices α6-α10 mentioned in the manuscript (probably there

are few residues with high correlation but there is no collective trend). Furthermore, There is no clear correlated motion between N-terminal and C-terminal domains (at least visually). Therefore, I do not understand the claim about the role of correlated motions in the communication of acetyl-CoA binding to the active site (Fig 3c does not help in this regard)."

The correlation analysis herein only considered linear correlations between the residue fluctuations during the equilibrated time period of the MD simulations. The Pearson product-moment correlation coefficients were computed in Python. We have updated the Methods section to include the details of how the correlation analysis was conducted.

We have also updated the correlation matrices by incorporating results from the additional simulations. The new correlation matrices clearly show two regions with high correlation values, the N-terminal ICL2-unique helical substructure (α10-α16, residues 278-427) and the C-terminal domain (residues 603-764). We have re-analyzed the updated correlation matrices in detail. As a result of including the additional simulations, the clear correlated motion pathway linking the ICL2-unique helices and the movements in the active site loop (as shown previously in Figure 3c) was no longer observed. We have updated the manuscript and the figure accordingly to reflect this new finding. We thank Reviewer #3 for the input that has improved this manuscript.

"3. In the manuscript, the calculation of free energy of assembly dissociation has been referred to the paper by Krissinel & Henrick. However, there is no indication about the exact way the free energy has been calculated (or the equation that has been used). To make the manuscript clear and reproducible, I request to add a section in the method and describe this calculation. On the other hand, considering the error in the calculation, the ΔΔG of ~1.5 Kcal/mol is probably a negligible difference."

This value was calculated by the PDBePISA server. We have now replaced these values with the buried interface area to better indicate the changes in the interface upon formation of the new dimer in the C-terminal domains.

"4. Another issue is the cryptic way of describing the structural search to find the similarities with the GNAT superfamily. As this part is a central part to the manuscript and the role of Acyl-CoA in the function of ICL2, it would be good also to add the exact description of the search protocol in the method section."

The C-terminal domain of ICL2 (residues 595-764) was compared against the structures in the Protein Data Bank (PDB) using the DALI server (http://ekhidna2.biocenter.helsinki.fi/dali/). The resulting results were ranked based on Z score, with the 4UA3 entry showing the highest Z score (10.4) in the list. The results were confirmed by the complementary 3D-BLAST Protein Structural Search online server (http://3d-blast.life.nctu.edu.tw/). We have added the information in the Experimental section.

"5. Supp Figure 11 has not been discussed in the text. However, it contains several interesting points. C-terminal domain in the apo state has a quite high RMSD, which probably means internal instability of this domain in absence of Acyl-CoA. Acetyl-CoA also has a quite high RMSD. Does such a high RMSD mean that the binding mode of Acetyl-CoA changes during the simulation? In particular between frame ~600-800 there is a sharp rise in the RMSD of Acetyl-CoA which probably requires an explanation."

The structures of ICL2 were first aligned at the N-terminal domain before the RMSD values were calculated. Therefore, the high RMSD values of the C-terminal domains in ligand-free ICL2 reflect the flexibilities of the whole C-terminal domain, and its movements relative to the N-terminal domain. Similarly, the fluctuations observed in the acetyl-CoA RMSD values were due to the movements of the C-terminal domain. When only the acetyl-CoA molecules in each chain are aligned, the RMSD values show that the binding conformations of acetyl-CoA are quite stable. We have updated SI Figure 14 to include the RMSD values of the new simulations. We have included only the protein backbone RMSD values for the whole enzymes in the updated figure as we wanted to show that the simulations have equilibrated.

"1. In general, the way of presenting figures and the coloring method could be improved."

We have taken this point on board and have updated a number of figures in the manuscript.

"2. Fig 3a is difficult to understand and also two Xray structures are not visible. It would be better to use transparent coloring."

We updated Figure 3a to include the new trajectories obtained. We have modified the colour of the schemes in this figure to improve clarity. We only included one crystal structure (3P0X) in this figure as the two crystal structures show identical conformations at the active site loop.

"3. In Supp Fig 9a and c, it is not clear how the regions with local changes were identified. For example, the region 88-92 in 9a does not seem to have high fluctuation (RMSF < 2)."

The regions with local changes were identified if the RMSF/RMSD average values from one system do not fall in the RMSF/RMSD ranges (average ± error) of the other system. We have updated supplementary Figure 12.

Reviewer #4

"Line 73 I would like to see the values for the buried surface areas, since the binding energies are inferred from these."

Please see response to Reviewer #3, point 3. The buried surface areas are as below:

Ligand-free: 975 Å^2
Acetyl-CoA-bound: 1105 Å^2

"Lines 84-88 I don't think this observation is sufficiently described. I am not clear how Figure 3a and b show this. 3P0X is identified in the Figure, but 1F8I is not."

We have expanded our descriptions of the conformational shift in the active site loop in the main text. Figure 3a and 3b were updated. There is only one crystal structure included in Figure 3a (3P0X) for clarity, as the both 3P0X and 1F8I show the same conformation at the active site loop.

"Line 93 These residue numbers can't be the helices - more residues are in the helices."

We have changed the manuscript according to the results from our updated analysis.

"Line 95 Figure 3c has ICL2 unique helices written in the upper left, but it is not clear to me which they are. They are not identified in the legend."

We have included additional simulations in our new analysis (see response to Reviewer #3), and the correlation pathway is no longer observed. We have removed Figure 3c.

"Figure 1's legend could be improved by explaining that the enzyme is a tetramer: The core is tetrameric, but the C-terminal domains dimerize. Although this is explained in the paper, many readers will look at the figures first."

Thank you for valid comment. We have updated the figure legends as suggested.

"In Figure 2, it is hard to see the error bars."

We have updated the figure error bars to make them more easily visualised.

"Figure 3b: I am not clear on this plot. The Figure title explains that the results from an MD simulation of ICL2 are shown. The x-axis has Calpha RMSD values - how are these so evenly spaced? Is it really a histogram? What are the number of values plotted on the y-axis? The plot is for 8 residues. Would it not be better to show RMSD values for the 8 residues? I am not clear whether the values on the left of the red plot are zero or one - but my confusion might be that I don't understand the "Number of values" plotted on the y-axis."

Figure 3b is plot of the distribution of RMSD values between the active site loop from each frame of trajectories in MD simulations and the crystal structure of isocitrate-bound ICL1 from *Brucella melitensis* (*Bm*-ICL1, PDB 3P0X). Plotting the distribution will give us an indication of the conformational population at the active site loop, the x-axis indicates bin centres from the distribution analysis, and the y-axis is the number of structures from MD simulations that contain RMSD values in the corresponding bin. Therefore, Figure 3b shows that active site loop samples different conformations in ligand-free and acetyl-CoA-bound ICL2. The active site loop in acetyl-CoA-bound ICL2 possesses similar conformations to that in substrate-bound *Bm*-ICL1, indicating of a conformational shift towards more catalytic relevant conformations upon binding of acetyl-CoA. We have updated Figure 3b incorporating results from the additional MD simulations.

"Figure 3c: Is the correlation in the movement of these residues?"

We no longer observe the correlated movements between these residues, so Figure 3c has been removed.

"Supplementary Figure 3: Is the additional helical substructure the part on the left? There are also two helices middle bottom in wheat. In the paper, the additional helices are alpha10-16. Perhaps labelling them here would help."

We have now updated the figure and have labelled the structure for clarity.

""Cartoon representation" is used where I would say ribbon diagram. I'm not sure if this terminology is from the program PYMOL."

We have used cartoon model based on pymol definitions.

"Supplementary Figure 6 a. Why are there no points before 4 min? Are curves fitted to the points and initial rates calculated from these? Initial rates would normally be measured from the linear portion. Is the error range on the red curve correct? It seems too low. The red and purple curves do not look that different. The error for the orange curve also seems low. What is Tris-D11?"

Many thanks for the reviewer's comments. We have double checked and reanalysed our data to ensure accuracy. The 4 minutes was the lag time that was required between sample preparation and acquiring the first spectrum in the NMR instrument. We have updated our figure legends to clarify this. The initial rates were extracted through non-linear curve fitting (for further details, please see response to Reviewer #1). Tris-D11 refers to deuterated Tris buffer, which is needed for ^{1}H NMR experiments so that the buffer peak will not dominate the spectrum.

"Supplementary Figure 7d does not look like a plot of Michaelis-Menten kinetics, yet there is no explanation of the S-shape."

Please see response to Reviewer #1

"Supplementary Figure 8. I cannot judge whether molecular dynamics calculations can confirm a water molecule. I would have thought that the coordination would distinguish water from Mg2+."

This is an excellent point. We believe the coordination does not resolve the issue due to the electron density of Mg^{2+} and oxygen in water are virtually the same. In addition, hydrogen atoms are not detected in the water, therefore the assignment between Mg^{2+} and H_2O is not straightforward. By testing the stability of the coordination bonds of the ion and the hydrogen-bonding pattern of the water molecule using MD, the two scenarios were tested in parallel. The results were quite clear that water formed stable hydrogen bonds within the protein crystal whereas the positive ion was ejected showing that the crystal matrix does not accommodate a highly charged entity in said position. These results clearly indicate a water molecule is present in this position and not Mg^{2+}

"Supplementary Figure 9a. I could not distinguish the plots. Am I correctly interpreting that the direction of the arrow, up or down, indicates which model shows greater flexibility in the simulation?"

Yes, the reviewer is correct. We have updated this figure to incorporate results from our new simulations.

"Supplementary Table 2. Are the data from ICL2-acetyl-CoA (form I) important? Although it is listed as a different crystal form, the space group and cell dimensions are similar to those of form II. Why not include only the better-diffracting crystal? My understanding from the protein crystallography is that the structure of the apo form was first determined at low resolution, 2.9 Å. When a crystal that diffracted to 1.8 Å was found, the model was refined against the higher resolution data set. This data set and the model are described in the tables. For the complex with acetyl-CoA, two crystal forms are described, but other than the difference in resolution, the models seemed the same. Is there any need to discuss the first crystal form for the complex?"

We have now removed the lower resolution acetyl-CoA bound structure from Supplementary Tables 2 and 3.

Reviewers' comments:

Reviewer #1 (Remarks to the Author):

The authors have addressed most of the concerns from the first round of review. However, there are a few remaining issues that should still be dealt with before publication is appropriate.

1. With regard to the concern of reaction reversibility and the extent of reaction observed by the authors, I would like to first apologize to the authors for miscalculating the concentration of succinate/glyoxylate that would be expected under their conditions. At equilibrium, there should be only about 10 μM D-isocitrate remaining. However, the statement that "complete conversion" had been obtained is not accurate for a reversible system like this. The authors should instead refer to the situation as "no detectable isocitrate" or something equivalent.

2. I find the authors' response to the sigmoidal kinetics exhibited in Supp. Fig. 7d (now labeled 8d) unsatisfactory. The authors claim, "We believe the relatively low readings at 25 μM and 50 μM propionyl-CoA are likely due to poor signal-to-noise as the rate of 2-methylisocitrate turnover was slow at low propionyl-CoA concentrations." The rates corresponding to these two points are <10 μM/min. In making their statement, the authors are essentially admitting that (1) their error bars grossly underestimate the actual error, and (2) the accuracy of any data with rates <10 μM/min is in question. All of their plots have 1 or 2 points near or less than this rate. If these points are omitted, however, poor estimates of Km would be obtained. Rather than dismissing the data that don't conform to a hyperbolic plot, the authors should be collecting all kinetic data with the UV-Vis assay, which is capable of accurately measuring these rates, unlike the NMR method. If the kinetic model does in fact involve allostery, that would make their results even more impactful.

Reviewer #2 (Remarks to the Author):

I have examined the point by point response letter and the revised manuscript, and I believe that the points raised in the previous round of review have been satisfactorily addressed by the authors.

Reviewer #3 (Remarks to the Author):

The revised manuscript by Bhusal et al., reporting the structure of Mycobacterium tuberculosis isocitrate lyase 2, has improved considerably compared to the initial version. Now the description of the findings in the main text is clear. Though, I still think the manuscript needs improvements, upon addressing the remaining issues I would recommend the publication.
1. The issue about sampling has not been addressed properly. Adding more simulations will help to be more confident about the results. However, as the conformational space is unknown and also the transition rates between the states are not determined, the authors could not be sure about the states that both Apo and acetyl-CoA-bound could sample. As an example, apo state did only sample one conformation but one can imagine that the second conformation is still possible for the apo state but the transition rate is in microsecond timescale, therefore in the current simulations' timescale the apo structure will not sample the other conformation and in a much longer simulation the distribution of conformations in Fig. 3b can change. That was the reason, I asked for the enhanced sampling methods (i.e. replica-exchange simulation, metadynamics, etc).
I still agree that current results imply the conformation of the active site loop is probably different in the apo and acetyl-CoA-bound states, but this should be presented as a possibility not a firm conclusion.
2. Also, this statement in both the response and the manuscript "As there are 4 identical protein chains in each simulation, effectively we have collected 828.4 ns x4=3313.6 ns = 3.3 μs of trajectories for ligand-free ICL2, and 705 ns x4= 2820 ns=2.8 μs of trajectories for acetyl-CoA-bound ICL2" is not correct. The dynamics of monomers in the tetramer is not independent and unless there is a clear sign that the dynamics of the active site loop in each monomer is not

affected by the adjacent protomers, such a multiplication of simulations' time is not valid!

Reviewer #1

The authors have addressed most of the concerns from the first round of review. However, there are a few remaining issues that should still be dealt with before publication is appropriate.

1. With regard to the concern of reaction reversibility and the extent of reaction observed by the authors, I would like to first apologize to the authors for miscalculating the concentration of succinate/glyoxylate that would be expected under their conditions. At equilibrium, there should be only about 10 μM D-isocitrate remaining. However, the statement that "complete conversion" had been obtained is not accurate for a reversible system like this. The authors should instead refer to the situation as "no detectable isocitrate" or something equivalent.

We thank the reviewer for the feedback. We agree with the reviewer that we should have phrase our statement as ‘no further detectable changes in isocitrate’ rather than ‘complete turnover’ in our previous ‘response to reviewer’ letter.

2. I find the authors' response to the sigmoidal kinetics exhibited in Supp. Fig. 7d (now labeled 8d) unsatisfactory. The authors claim, "We believe the relatively low readings at 25 μM and 50 μM propionyl-CoA are likely due to poor signal-to-noise as the rate of 2-methylisocitrate turnover was slow at low propionyl-CoA concentrations." The rates corresponding to these two points are <10 μM/min. In making their statement, the authors are essentially admitting that (1) their error bars grossly underestimate the actual error, and (2) the accuracy of any data with rates <10 μM/min is in question. All of their plots have 1 or 2 points near or less than this rate. If these points are omitted, however, poor estimates of Km would be obtained. Rather than dismissing the data that don't conform to a hyperbolic plot, the authors should be collecting all kinetic data with the UV-Vis assay, which is capable of accurately measuring these rates, unlike the NMR method. If the kinetic model does in fact involve allostery, that would make their results even more impactful.

We thank the reviewer for the feedback. As recommended by the reviewer, we have repeated the measurements by UV/vis assay. The data showed that the data is not sigmoidal. The data is included as Supplementary Figure 8.

Reviewer #2

I have examined the point by point response letter and the revised manuscript, and I believe that the points raised in the previous round of review have been satisfactorily addressed by the authors.

We thank the reviewer for the help with this manuscript.

Reviewer #3

The revised manuscript by Bhusal et al., reporting the structure of Mycobacterium tuberculosis isocitrate lyase 2, has improved considerably compared to the initial version. Now the description of the findings in the main text is clear. Though, I still think the manuscript needs improvements, upon addressing the remaining issues I would recommend the publication.
1. The issue about sampling has not been addressed properly. Adding more simulations will help to be more confident about the results. However, as the conformational space is unknown and also the transition rates between the states are not determined, the authors could not be sure about the states that both Apo and acetyl-CoA-bound could sample. As an example, apo state did only sample one conformation but one can imagine that the second conformation is still possible for the apo state but the transition rate is in microsecond timescale, therefore in the current simulations’ timescale the apo structure will not sample the other conformation and in a much longer simulation the distribution of conformations in Fig. 3b can change. That was the reason, I asked for the enhanced sampling methods (i.e. replica-exchange simulation, metadynamics, etc).
I still agree that current results imply the conformation of the active site loop is probably different in the apo and acetyl-CoA-bound states, but this should be presented as a possibility not a firm conclusion.

We thank the reviewer for raising this point. Indeed, the limitation in sampling during conventional MD simulations has been noted in literature. "How long is long enough for a MD simulation?" remains a question that affect all computational biochemists. In general, the sampling may be improved by either extending the equilibrated MD simulations to a much longer time scale (which is what we have done here), or by running simulations with enhanced sampling algorithms (as suggested by the reviewer). We believe our extended and additional simulations are sufficient for the purpose of this study and simulation results suggest a difference in active site loop conformational populations between the apo and acetyl-CoA-bound ICL2 systems, which the reviewer also agrees. Nevertheless we have conducted replica exchange MD (REMD) calculations for both ligand-free and acetyl-CoA-bound states of ICL2. Two sets of REMD simulations were set up. The first set consists 10 replicas exchanging between temperature range 300-310 K, which collected ~50 ns of trajectories per replica for each of ligand-free and acetyl-CoA-bound systems. The second set consists of 10 replicas exchanging between temperature range 310-320 K, which we have collected ~22 ns per replica. So far we have not observed any more sampling of the active site loop conformations in REMD than that in the conventional MD simulations, and the population of the active site loop remains different between the apo state and the AcCoA bound active state of ICL2.

The conformational landscape for a protein is vast and complex, thus we acknowledge this reviewer's concern and have re-written sentences in the MD section to "soften" claims made based on MD results. We have also added our new REMD results in the supplementary (Supplementary Fig. 14).

2. Also, this statement in both the response and the manuscript "As there are 4 identical protein chains in each simulation, effectively we have collected 828.4 ns x4=3313.6 ns = 3.3 μs of trajectories for ligand-free ICL2, and 705 ns x4= 2820 ns=2.8 μs of trajectories for acetyl-CoA-bound ICL2" is not correct. The dynamics of monomers in the tetramer is not independent and unless there is a clear sign that the dynamics of the active site loop in each monomer is not affected by the adjacent protomers, such a multiplication of simulations' time is not valid!

This is a very interesting point. We agree with the reviewer that the 4 monomers in ICL2 tetramer are not moving independently from their adjacent protomers. However, the multiplication of simulation time does not require the monomers to be independent to hold valid, it requires the monomers to be indistinguishable from each other to be valid. ICL2 is a homotetramer, and superimposition of individual chains show that each chain adopts the same conformation. Furthermore, the interfaces between the monomers are identical. For example, the N-terminal domain of chain A forms interfaces with N-terminal domains of chains B, C and D; this interface is identical to the interface formed between N-terminal domain of chain B and those of chains A, C and D. The tetramer superimposed using different chains as references will still completely overlay. Therefore, we cannot distinguish the four chains in ICL2 tetramer based on sequence, interfaces, or interactions. During MD simulations, each monomer's motion is under the same influence by their corresponding adjacent monomers. The same influence from adjacent monomers may result in different possible motions in the four chains and that is what we are sampling during the MD simulation. Thus effectively we are sampling the same scenario four times when we simulate the tetramer, which brings our collected trajectories to microsecond time scale. On the other hand, multiplication of simulation time will not be valid for protein hetero-multimers or proteins with non-identical interfaces between monomers. We also note that similar claims for simulation time have also been made in previous studies (*J Am Chem Soc*. 138(6): pp 2036-45, *J. Mol. Biol.*, 415: pp 716–726, and *Biophys. J.*, 116(10): pp 1887-1897).

REVIEWERS' COMMENTS:

Reviewer #1 (Remarks to the Author):

The authors have adequately addressed all concerns. Recommend publication.

Reviewer #3 (Remarks to the Author):

Overall the changes look sensible and the authors have address all the comments.
Just a minor typo in the caption of Supplementary Figure 14:
Last line should be
(a) high temperature range REMD (310K-320K) and (b) low temperature range REMD (300K-310K).

Otherwise, I do not see any further impediments to publication.
